# All-plasmonic Optical Phased Array Integrated on a Thin-film Platform

**DOI:** 10.1038/s41598-017-10398-8

**Published:** 2017-08-30

**Authors:** Yuan-Song Zeng, Shi-Wei Qu, Bao-Jie Chen, Chi Hou Chan

**Affiliations:** 1 School of Electronic Engineering, University of Electronic Science and Technology of China (UESTC), Chengdu, 611731 China; 2 State Key Laboratory of Millimeter Waves, Partner Laboratory in City University of Hong Kong, Kowloon, Hong Kong China

## Abstract

Optical phased arrays have been demonstrated to enable a variety of applications ranging from high-speed on-chip communications to vertical surface emitting lasers. Despite the prosperities of the researches on optical phased arrays, presently, the reported designs of optical phased arrays are based on silicon photonics while plasmonic-based optical phased arrays have not been demonstrated yet. In this paper, a passive plasmonic optical phased array is proposed and experimentally demonstrated. The beam of the proposed plasmonic optical phased array is steerable in the far-field area and a high directivity can be achieved. In addition, radio frequency phased array theory is demonstrated to be applicable to the description of the coupling conditions of the delocalized surface plasmons in optical phased arrays and thus the gap between the phased arrays at two distinctly different wavelengths can be bridged. The potential applications of the proposed plasmonic phased arrays include on-chip optical wireless nanolinks, optical interconnections and integrated plasmonic lasers.

## Introduction

Surface plasmons, which are the collective resonance of electrons on metal surface, have drawn a great attention in the field of nanophotonics due to their unique abilities to concentrate light beyond the diffraction limit^1^. In the past decade, the researches on surface plasmons, i.e., plasmonics, have been demonstrated to enable a variety of new technologies and applications^2^. Among them, plasmonic optical integrated chip (OIC) technologies offer the potential to achieve high levels of integration and miniaturization in OICs, and thus hold promise for future OIC technologies^3^. In recent years, many kinds of plasmonic waveguides^4–6^ and waveguide-based devices^7–9^ have been proposed and investigated for future plasmonic OIC technologies.

Optical phased arrays (OPAs), in which the interference of the emissions from a number of nanoantennas are utilized to focus and steer light^10^, have been demonstrated to have a promising prospect for OIC applications, e.g., 3-dimentional (3D) optical interconnections^11^, on-chip imagers^12^, vertical surface emitting lasers^13^, high-speed on-chip communications^14^ and vector beam generations^15^, to name just a few. In an OPA, the phase of each nanoantenna element needs to be properly aligned by wave-guiding structures so that a desired far-field radiation pattern can be formed. Although there are ongoing researches on nanoantennas fed by plasmonic waveguides^16–22^, the existing designs fall short of the freedoms to align the phases of the nanoantenna elements in an OPA. Moreover, the lossy nature of the surface plasmons also presents challenges in designing high-efficiency feeding networks for OPAs. Meanwhile, the experimental demonstrations of the existing plasmonic-waveguide-fed nanoantennas still rely on free-space-light-in and free-space-light-out scheme, which is not suitable for OIC applications. As a consequence, the existing demonstrations of OPAs are based on silicon photonics while plasmonic-based OPAs have not been demonstrated yet.

We herein propose and experimentally demonstrate a novel all-plasmonic OPA. The proposed plasmonic optical phased array (POPA) consists of thousands of plasmonic nanopatch antennas and a plasmonic nanostrip waveguide array which acts as the feeding network enabling the phase adjustments of the nanopatch elements. The geometry of the proposed POPAs is fully planar with a sub-micron profile and the light from a fiber can be directly coupled into the plasmonic nanostrip waveguide array. The beam of the proposed POPA is steerable and a high directivity in the far-field area can be achieved. Moreover, compared to silicon-based OPAs where a considerable part of power is emitted into the substrate layer^10, 15^ (the upward and downward emissions are at the same level), the proposed POPA is free of the energy leakage into the substrate side. These features make the proposed POPA a promising building block for plasmonic IC applications such as the construction of on-chip optical wireless nanolinks^22^, integrated plasmonic lasers^23, 24^ and nanoantenna couplers between photons and plasmonic integrated circuits^20, 25, 26^. Furthermore, considering the plasmonic nature of the proposed POPA, the influence of the delocalized surface plasmons^27–31^ on the proposed POPA is investigated. The results indicate that radio frequency (RF) phased array theory can be adopted for the description of the coupling condition of the delocalized surface plasmons in the proposed POPA. With the same array topology, a flat focusing POPA for 3D inter-chip interconnections is also demonstrated in the Disscussion section.

## Results and Design Principles

### Plasmonic nano-waveguide and nanoantenna

Existing candidate plasmonic waveguides to feed nanoantennas include surface plasmon channel^16^, surface plasmon gap structure^20^, surface plasmon nanowire^18^ and surface plasmon stripe^19^. However, coupling light into these structures is always a problem due to the significant size and momentum mismatches^32^ between these surface plasmon waveguides (SPWs) and conventional photonic components. As a result, special techniques are required to excite propagating plasmons in these SPWs^33–36^. In addition, these structures are unsuitable for the manipulation of a nanoantenna array, considering their highly confined fields and the lossy nature of plasmons. The above limitations prompt us to seek for an alternative for the POPAs.

**Figure 1:**
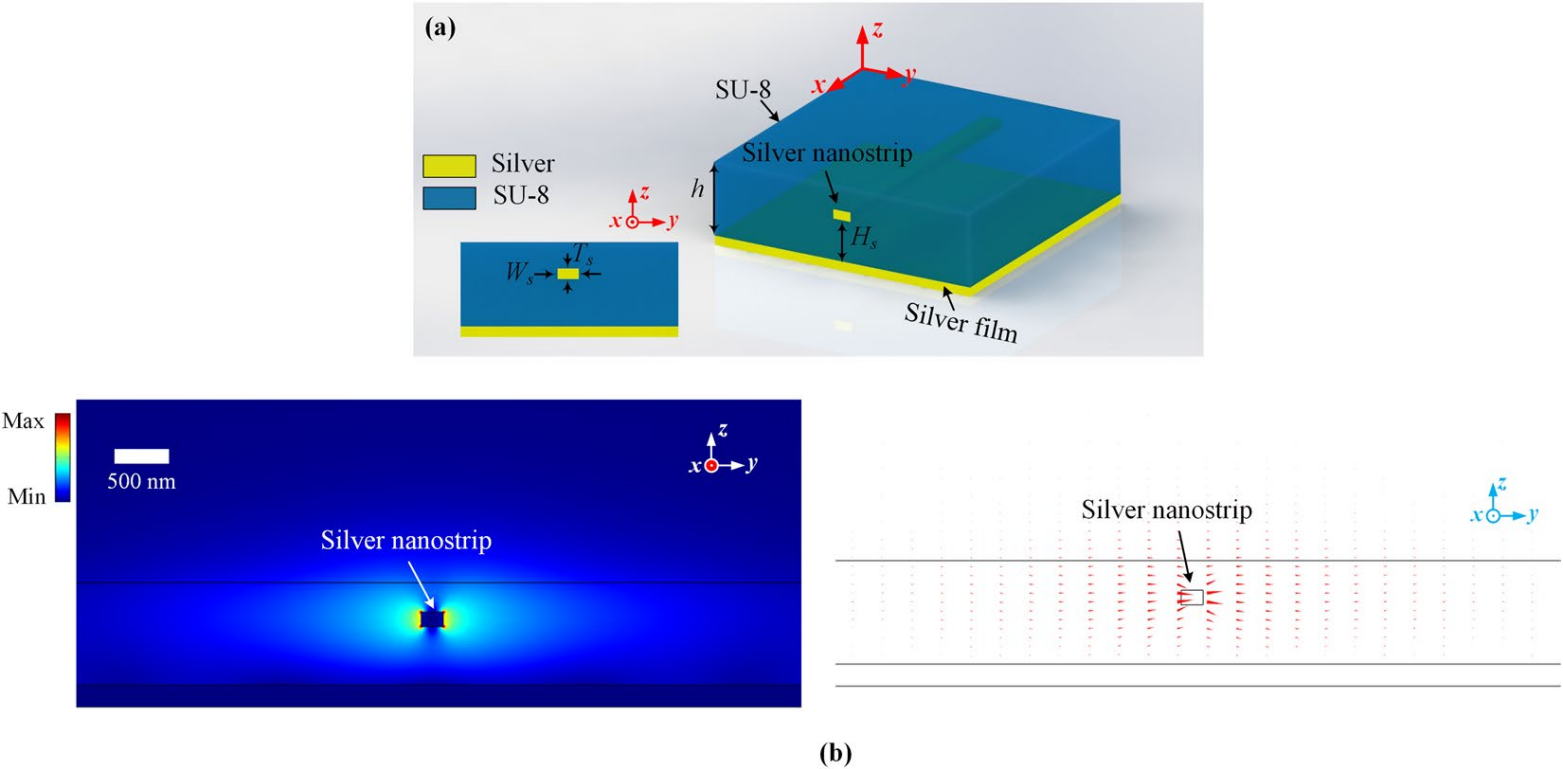
Proposed nanostrip SPW. (**a)** Geometrical sketch. (**b)** Electric field distributions on a cross section of (**a**) nanostrip SPW with *W*_*s*_ = 150, *T*_*s*_ = 100, *H*_*s*_ = 400 and *h* = 700 in nm.

The geometric sketch of the proposed SPW is shown in Fig. 1a. It is composed of a silver nanostrip, a SU-8 slab supporting the silver nanostrip and a silver film on the backside of the SU-8 slab. A plasmonic mode formed by hybridizing *ss*_*b*_^0^ mode of a nanostrip^37^ and surface plasmon polaritons (SPPs) on a metal-dielectric interface is supported by this waveguide. The nomenclature *ss*_*b*_^0^ is used to identify the mode symmetry with respect to the *z* and *y* axes. Detailed information about this nomenclature can be found in Section 1 of Supplementary Information. The electric field distributions of this mode at a cross section of the proposed SPW is depicted in Fig. 1b. The mode offers a significantly lower transmission loss than conventional SPWs (Supplementary Table S2) and thus it is attractive for the construction of a POPA. Detailed information of this SPW can be found in Section 1 of Supplementary Information.

**Figure 2:**
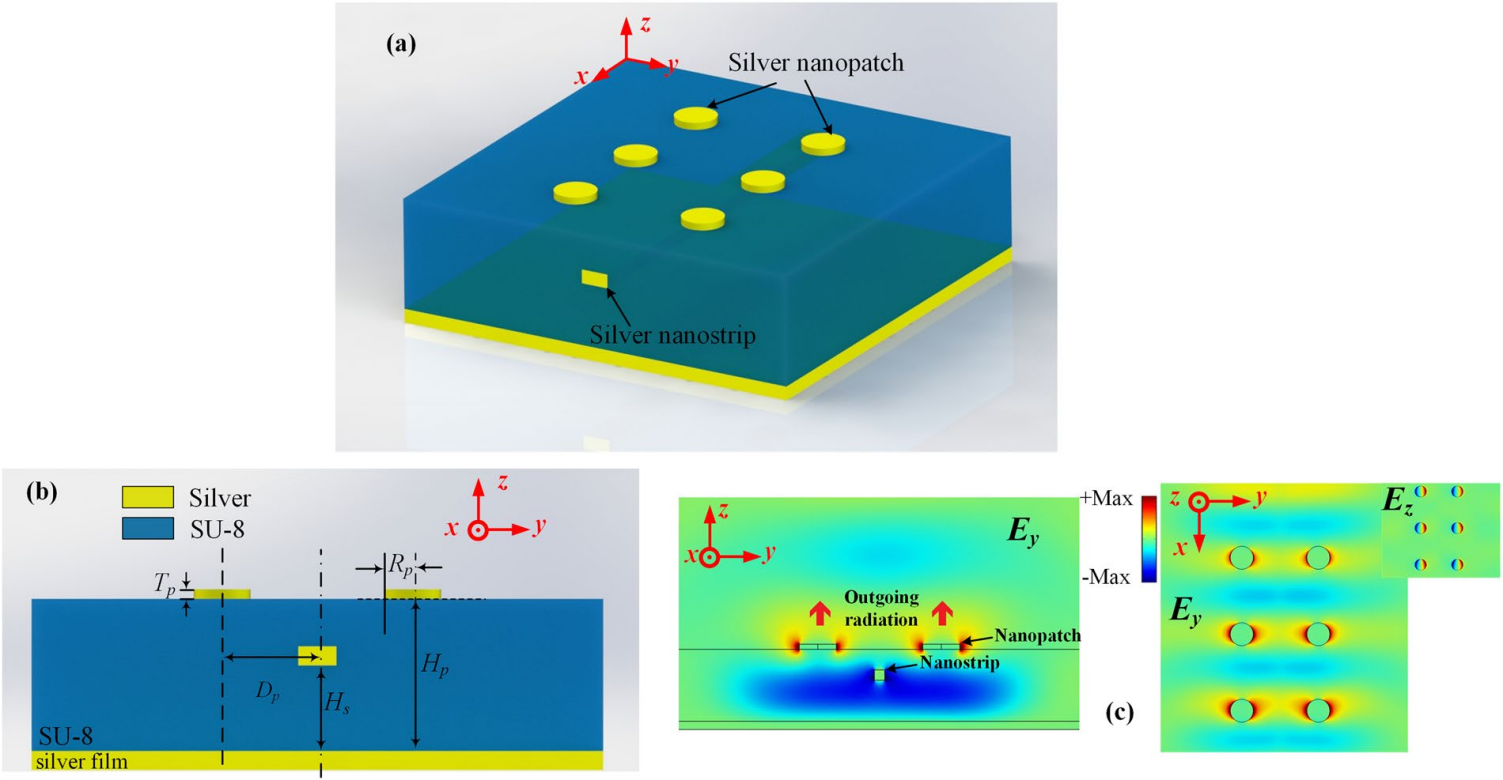
Capacitive coupling between nanostrip and nanopatches. (**a,b)** Schematics showing the position of the nanopatches and nanostrip. The coupling coefficient can be controlled by the distance between nanopatch and nanostrip which is denoted by *D*_*p*_ in (**b)**. (**c)** Left panel: *E*_*y*_ distribution on the *yoz* plane, at a certain phase, showing the outgoing radiation. The animated result can be found in Supplementary Materials (Supplementary Video S1). Right panel: *E*_*y*_ and *E*_*z*_ distributions on the surface of the polymer film, i.e. the lower surface of the nanopatches. The parameters are as follows: *H*_*s*_ = 400, *H*_*p*_ = *h* = 700, *W*_*s*_ = *T*_*s*_ = 100, *R*_*p*_ = 180, *D*_*p*_ = 600 in nm and the distance between the nanopatches in the *x-*direction is 1.2μm. The results are obtained at 1550 nm.

The plasmonic nanopatch antenna was first proposed by R. Esteban^38^ and then investigated for applications like single photon emission^39^, spontaneous emission controlling^40^, optical polarization converter^41^ and holograms^42^. Here, near-field interactions between the nanopatch and the nanostrip are exploited to excite the fundamental mode of the nanopatch. As depicted in Fig. 2, when the nanopatches are in proximity to the nanostrip, the localized surface plasmons (LSPs) on the nanopatches will be excited by the displacement currents around the nanostrip via capacitive coupling. The LSP intensities on the nanopatches are determined by the distance *D*_*p*_, while the phase distribution among them can be controlled by the positions of the nanopatches along the *x*-axis.

Depending on the height *H*_*p*_ over the silver film, the operating mechanism of the nanopatch antenna can be either interpreted by the widely adopted cavity model^43^ or a nanoparticle backed by a plasmonic reflector when *H*_*p*_ is too large to sustain a cavity mode. The electric field distributions are presented in Fig. 2c to show the coupling behavior, where *E*_*z*_ is induced by the LSPs and confined near the nanopatches while *E*_*y*_ directly contributes to the emission. Due to the existence of the silver film, the potential energy leakage towards the substrate side is prohibited.

### Proposed POPA

**Figure 3:**
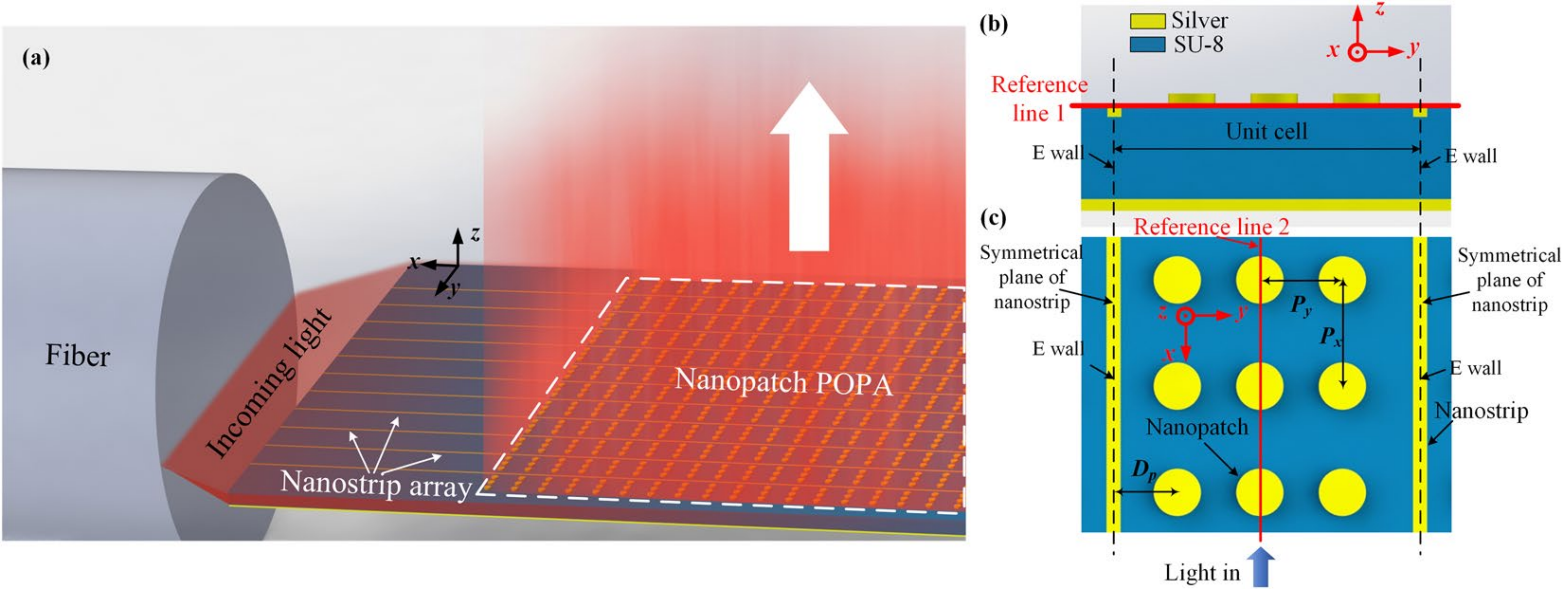
(**a**) Schematic showing the working mechanisms of the proposed POPA (not to scale). Light from a fiber is directly coupled into Nanostrip SPWs which act as a power relay which mediates power transfer from the fiber to nanopatches. (**b** and **c**) Schematics showing the unit cell design strategy on the *yoz* plane (**b**) and *xoy* plane (**c**). *P*_*x*_ and *P*_*y*_ represent the period in the *x-* and *y-* directions, respectively and *D*_*p*_ in this figure denotes the distance between the nanostrip and its neighbouring nanopatch.

The overall configuration of the proposed POPA is depicted in Fig. 3a. The nanostrip SPWs are arranged in parallel to the *x*-axis into an array to directly couple light from a fiber and also act as a power relay from the fiber to the nanopatches. The nanopatches then convert the energy from the nanostrip SPWs back into photons. The dimensions and relative positions of the nanopatches inside each unit cell of the nanostrip array are depicted in Fig. 3b and c.

Note that the *y*-polarized fields of the proposed nanostrip SPW have a natural symmetry with respect to the *xoz* plane and the electric field intensity is dominated by the *y* component (Supplementary Fig. S2). Benefiting from the two aforementioned unique modal properties, when the nanostrips are arranged together into an array, there is an in-phase superposed mode which is free of obvious distortions over the original mode characteristics of the nanostrip SPW (Supplementary Information Section 1.6). Moreover, by arranging the nanostrips into an array, the modal size of the nanostrip SPW is equivalently enlarged and the significant size mismatch between the SPW and the fiber is diminished.

Considering the aperture size of the fiber, a large area containing numerous nanostrips will be covered by the fiber. Usually it is impractical to obtain the solution of such an electrically large structure directly with full-wave simulations. However, due to the periodic nature of this case, the calculation domain can be reduced by applying periodic boundary conditions in a unit cell. Here, electric walls (*E*_//_ = 0, d*E*_⊥_/d*n* = 0, *H*_⊥_ = 0, d*H*_//_/d*n* = 0) are employed at the symmetrical planes of the adjacent nanostrips for simplicity, as depicted in Fig. 3b and c.

**Figure 4:**
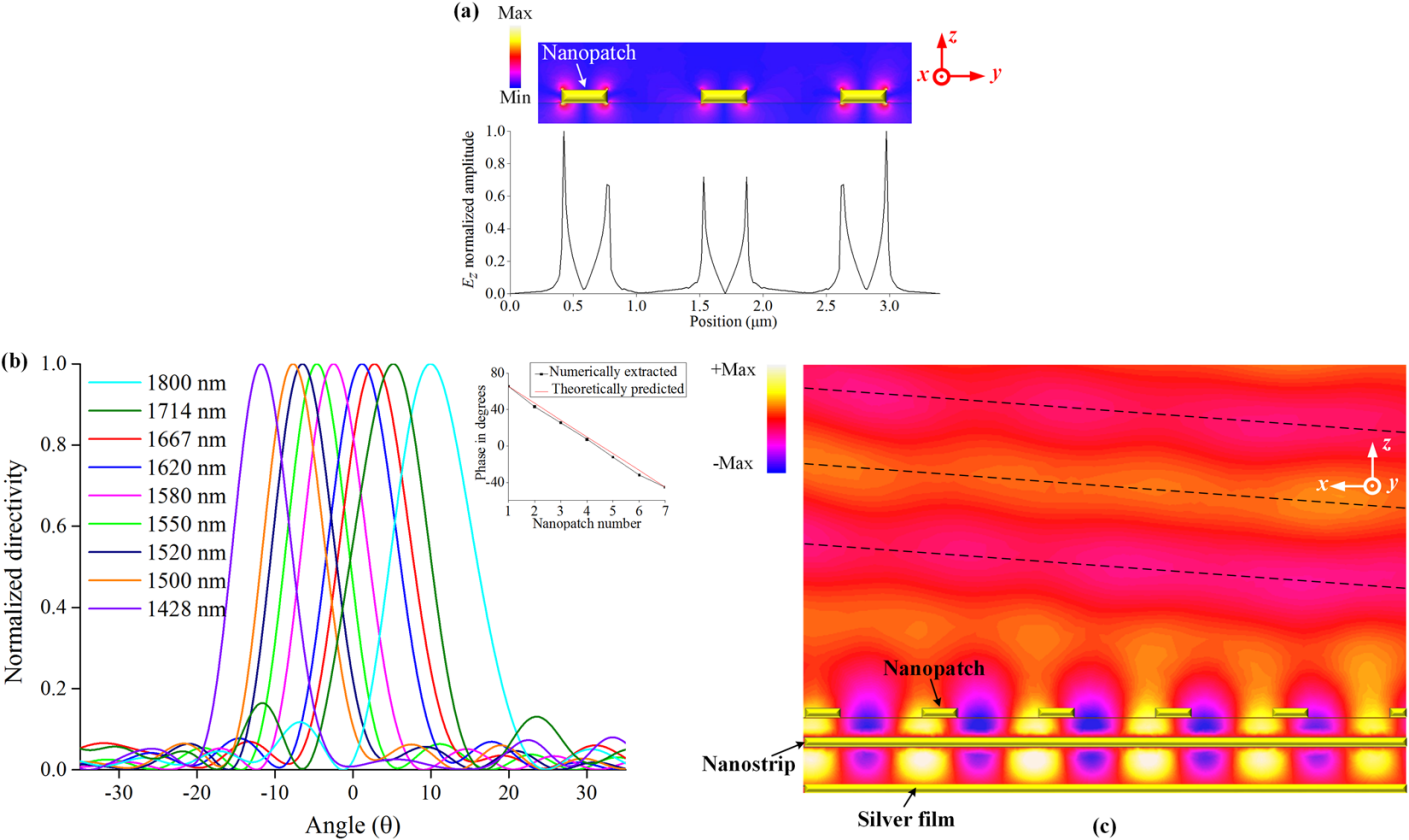
Near-field and far-field results of the proposed POPA. (**a)** The picture with colorbar depicts the intensity of *E*_*z*_ on the *yoz* plane. The curve is the intensity of *E*_*z*_ along the reference line 1 in Fig. 3b, i.e. along the bottom surfaces of the nanopatches. (**b)** Simulated normalized far-field directivity patterns at various wavelengths. The inset shows the comparison between the theoretically predicted and the numerically extracted phases of the radiated component, i.e. *E*_*y*_ component along the reference line 2 in Fig. 3c. The reference line 2 is 50 nm above the upper surfaces of the nanopatches. (**c)** Near-field distribution of *E*_*y*_ in the central plane of the array showing the forming of a directional power emission which originates from the coherent interference of the nanopatches. The central plane is the symmetry plane of the array and in parallel with the *xoz* plane. The results are obtained at 1550 nm. The animated result can be found in Supplementary Materials (Supplementary Video S2).

For demonstration purpose, a unit cell with 7 × 3 nanopatches is numerically investigated. The geometrical paramaters of the investigated POPA are as follows: *W*_*s*_ = 100, *T*_*s*_ = 100, *H*_*s*_ = 400, *h* = 700, *R*_*p*_ = 180, *T*_*p*_ = 100, *H*_*p*_ = 700, *D*_*p*_ = 600, *P*_*x*_ = 1200 and *P*_*y*_ = 1100 in nm. Note that depending on the distance between the adjacent nanostrips, there is a trade-off between the uniformity of the intensities of the excited LSPs along the *y*-axis and the amount of the nanopatches that can be covered within each unit cell (Supplementary Fig. S9). A 3.4 μm distance is selected here to reach a compromise between them. As an indicator of the LSPs intensities, the intensities of *E*_*z*_ on the bottom surfaces of the nanopatches are monitored along the reference line 1 in Fig. 3b. As seen from Fig. 4a, the intensities of the LSPs on three nanopatches are on the same level. With a uniform spacing along the *x-*axis, the nanopatches in the POPA are expected to exhibit a gradient phase variation along the positive *x-*axis given by *φ*_*step*_ = 2π (*P*_*x*_ − *λ*_*g*_)/*λ*_*g*_, where *λ*_*g*_ is the guided wavelength of the nanostrips (λg = λ0 / neff, where λ0 is the free space wavelength and neff is the effective mode index of the nanostrip SPW). The numerically extracted phases of Ey component, i.e., the fields contributing to emission, on seven nanopatches along the *x-*axis are compared with the theoretically predicted phases in the inset of Fig. 4b. The reasonable agreement between them demonstrates the ability of the nanostrip to manipulate the phase of the nanopatches. With a gradient phase variation along the *x-*axis, the emissions of these nanopatches are expected to interfere constructively at a certain angle. The near-field distribution of the investigated POPA in the *xoz* plane is plotted in Fig. 4c. It clearly indicates that a coherent interference of the radiated power from the nanopatches can shape the far-field emission pattern into a directional one. The simulated far-field emission patterns in the *xoz* plane are shown in Fig. 4b. Five of the telecommunication windows (*E, S, C, L and U* bands) are covered by the investigated POPA with an access up to 1800nm. When the wavelength increases from 1428 nm to 1800 nm, the beam experiences a scanning from the backward to the forward region, covering an angular range of 21.7 degrees.

### Impacts of the delocalized surface plasmons

Another key issue that needs to be taken into account is the influence of the delocalized surface plasmons^27–31^. Different from the non-propagating LSPs, the delocalized surface plasmons are the propagating electromagnetic waves bounded on the metal-dielectric interface, also known as propagating surface plasmons (PSPs). In certain circumstances, the PSPs can be excited by the additional momentum provided by the periodicity of the nanoparticle array. The interaction between LSPs and PSPs under plane wave illumination has been widely explored^44–46^ and it has been shown that their mutual coupling will result in a shift of the resonant wavelength of the LSPs^27^ or an anti-crossing behavior of the resonant wavelengths of the LSPs and PSPs^45^.

**Figure 5:**
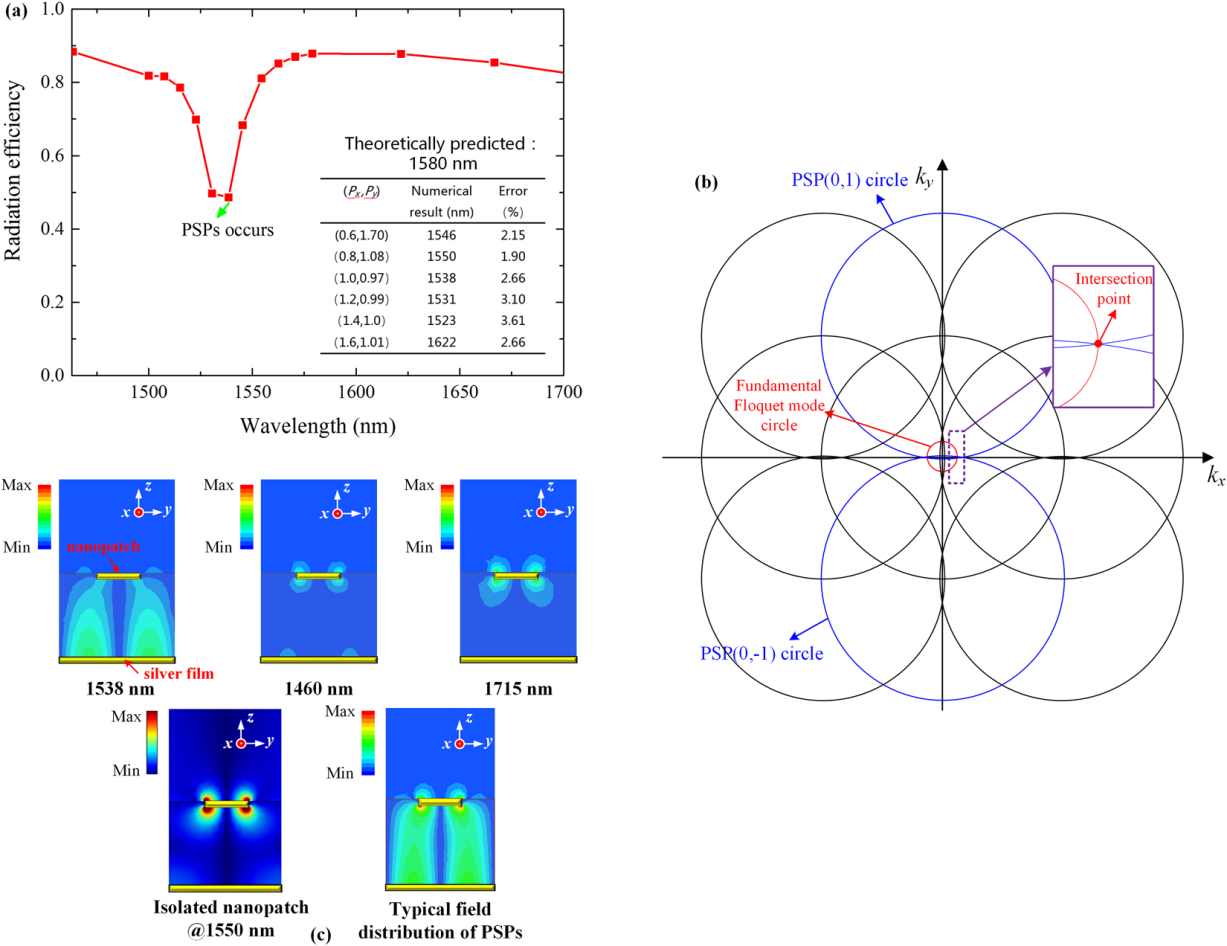
Coupling between the fundamental Floquet mode and the PSP modes. (**a)** Radiation efficiency of the investigated POPA. Array configurations are as follows: *H*_*s*_ = 600, *H*_*p*_ = 650, *h* = 700, *W*_*s*_ = *T*_s_ = 100, *R*_*p*_ = 180, *D*_*p*_ = 500, *T*_*p*_ = 50, *P*_*x*_ = 1000 and *P*_*y*_ = 970 in nm. A unit cell containing 21 nanopatches (7 in row and 3 in column) is simulated. The table shows the comparison between the numerically obtained coupling wavelengths and the theoretically predicted ones. (**b)** Floquet circle diagram indicating the coupling of the fundamental Floquet mode and the PSP (0, ±1) mode in the *k*-space at 1580 nm. The red circle is the fundamental Floquet mode circle while the blue and black ones are the PSP circles. The inset shows the zoom-in view of their intersection point. (**c)** Intensity of the *E*_*z*_ component on a reference plane that is in parallel with the *yoz* plane and passing through the center of the nanopatches. Upper-half panel, from left to right: intensity of the *E*_*z*_ component at the wavelength where PSPs are predicted to occur (1538 nm), that at 1460 nm and 1715 nm. Lower-half panel, from left to right: intensity of the *E*_*z*_ component of the isolated resonant nanopatch and that at the PSP-emergence wavelength when the same array is illuminated by plane waves (Supplementary Information Section 4).

For the investigated POPA, numerical results indicate that the coupling condition of the PSPs can be described by the interaction between the Floquet modes and the PSP modes^47^:

1 $${({k}_{xmn}-\frac{2(p+m)\pi }{{P}_{x}})}^{2}+{({k}_{ymn}-\frac{2(q+n)\pi }{{P}_{y}})}^{2}={\beta }_{psp}^{2}\quad (m,n,p,q\,{\rm{are}}\,{\rm{integers}})$$

where *m, n* denote the orders of the Floquet modes and *p, q* denote the orders of the PSPs in the *x-* and *y-* directions. In equation (1), *k*_*xmn*_ and *k*_*ymn*_ denote the locations the (*m*, *n*) Floquet circle in the *k* space and *βpsp* is the phase constant of the PSPs (${\beta }_{psp}={k}_{0}\sqrt{{\varepsilon }_{m}\,\ast \,{\varepsilon }_{d}/({\varepsilon }_{m}+{\varepsilon }_{d})}$, where *k0* is the free-space wavenumber, *εd* is the dielectric constant of SU-8 and *εm* is the dielectric constant of silver). As the high-order Floquet modes will give rise to the grating lobes or aliased lobes in the far-field^47, 48^, only the fundamental Floquet mode (*m* = *n* = 0) is of our concern47. Hence equation (1) can be simplified to:

2 $${({k}_{x0}-\frac{2p\pi }{{P}_{x}})}^{2}+{({k}_{y0}-\frac{2q\pi }{{P}_{y}})}^{2}={\beta }_{psp}^{2}$$

where *k*_*x*0_ is given by *k*_*x*0_ = 2π (*P*_*x*_ − *λ*_*g*_)/(*P*_*x*_ *λ*_*g*_) and *k*_*y*0_ is equal to zero for the investigated POPA. The comparison between the theoretically predicted coupling wavelengths and the ones obtained by numerical simulations is shown in the inset of Fig. 5a. Taking the case with *Px* = 1 μm and Py = 0.97 μm as an example, in such a configuration, the PSP (0, ±1) (p = 0, q =  ±1) modes (the index numbers are assigned in the order of x- and y- directions, respectively) are predicted at 1580 nm according to equation (2). Accordingly, in the full-wave simulations, the coupling is observed to occur at 1538 nm, showing an error of 2.66%. To provide a clear insight into this issue, the Floquet circle diagram associated with the investigated case (*P*_*x*_ = 1 μm, *P*_*y*_ = 0.97 μm) is plotted in Fig. 5b. The intersection of the fundamental Floquet mode circle and the PSPs (0, ±1) circle clearly indicates that their coupling condition is fulfilled in the investigated case.

In Fig. 5c, the field distributions of the investigated POPA at the coupling wavelength as well as two other wavelengths are depicted. An enhanced interaction between the nanopatch and the silver film via *E*_*z*_ is clearly observed at the predicted wavelength. Comparatively, at 1460 nm and 1715 nm, this strong dependence on *E*_*z*_ is not observed and the distribution is very close to that of the isolated nanopatch (see the first figure of the lower-half panel in Fig. 5c) where *E*_*z*_ is locally sustained by the LSPs around the nanopatch and the SPPs over the silver film, respectively.

For further verification, the results are also compared with the far-field illumination case in which the principles of light-PSPs coupling has been experimentally demonstrated^45^. The field distribution at the theoretically predicted coupling wavelength under the plane wave illuminations is depicted in the second figure of the lower-half panel in Fig. 5c, and it is found to be highly consistent with what has been previously observed in the investigated POPA. This consistency provides a solid evidence of the emergence of the PSP mode.

A side effect resulting from the emergence of the PSPs is a significant drop of the radiation efficiency. In Fig. 5a, the simulated radiation efficiency versus wavelength is plotted. With the emergence of PSPs, the POPA radiation efficiency drops by about 50 percent compared to that at 1460 nm (see Supplementary Information Section 2 for the calculation method of the radiation efficiency). This is not counter-intuitive because as the PSPs occur, a part of the energy is transferred into the SPPs on the silver film and barely contributes to the emission. The discussion on the resonant wavelength anomaly for the investigated POPA can be found in Supplementary Information Section 5.

Considering the conditions of no aliasing in the far-field at the telecommunication wavelength (1550 nm) and the size of each nanopatch when the LSPs are in resonance, the above discussion just makes sense when both *P*_*x*_ and *P*_*y*_ fall into the range between 0.5 μm and 1.6 μm. Under this restriction, the calculated results according to equation (2) indicate that the coupling between the fundamental Floquet mode and only four PSP modes with indices (0, ±1) and (−1, ±1) are possible. The calculated results at various wavelengths close to 1550 nm for PSP modes (0, ±1) and (−1, ±1) are plotted in Supplementary Figs S10a and S10b, respectively.

### Fabrication and measurement

**Figure 6:**
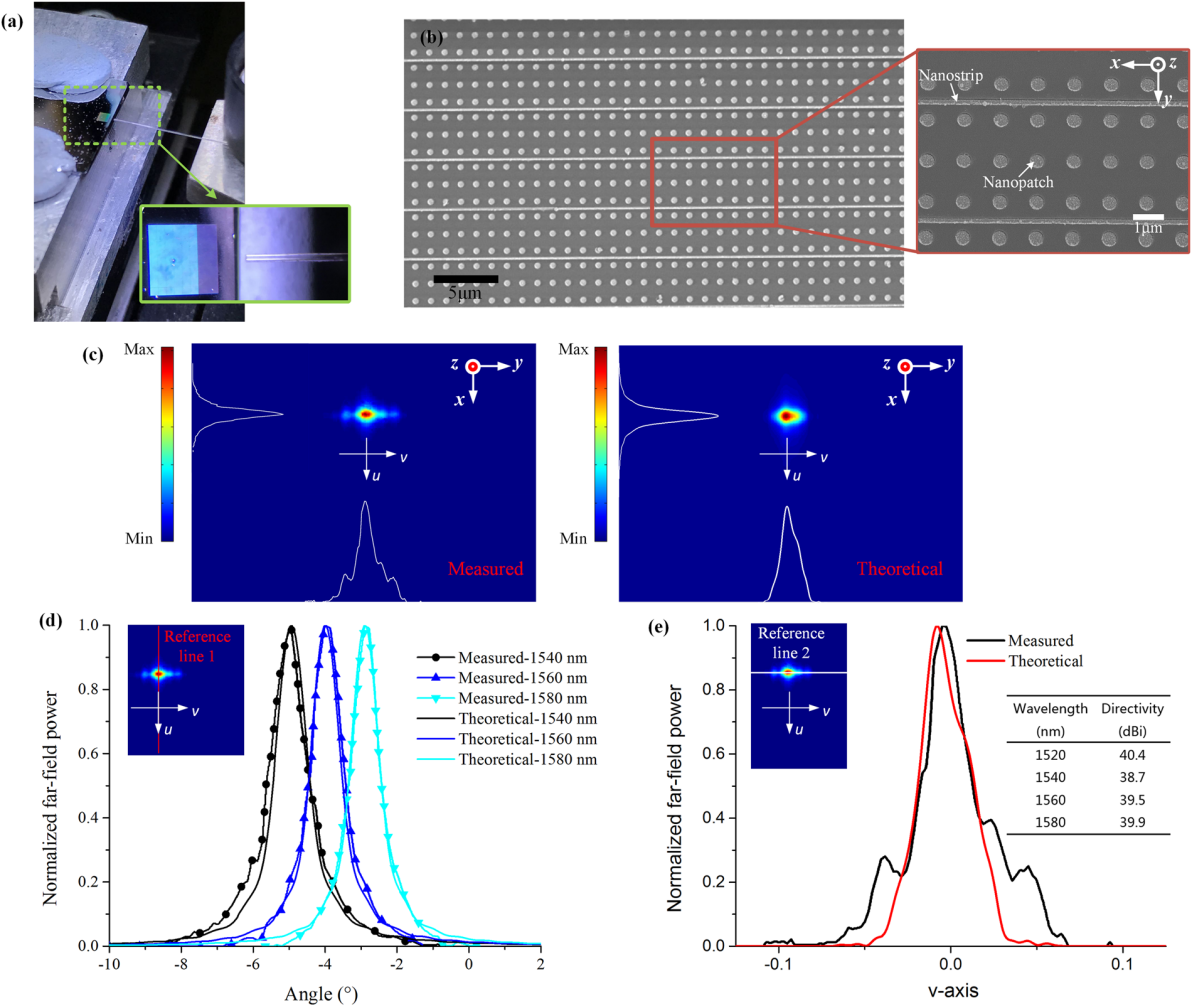
A large-scale POPA that is fabricated and experimentally characterized. (**a)** Image of the fabricated sample under microscopy showing the excitation via a fiber. (**b)** SEM images of the fabricated sample. (**c)** Measured (left one) and theoretically calculated (right one) far-field power emission patterns at 1540 nm. Both figures share the same coordinate ranges in the *u*-*v* space (*u* = sin(*θ)*cos(*φ*), *v* = sin(*θ)*sin(*φ*)), i.e. *v* from −0.283 to 0.283, *u* from −0.203 to 0.2205. (**d)** Measured and theoretically calculated far-field power profiles along the reference line 1 at various wavelengths, indicating a clear scanning behavior. (**e**) Measured and theoretically calculated power profiles along the reference line 2 at 1540 nm. The inset shows the theoretically calculated directivity of the fabricated POPA. The results in (**c**,**d** and **e)** are obtained under the quadratic phase distribution of *φ*_*n*_ with *a* = −0.125 (Supplementary Information Section 8).

Based on the calculation results in Supplementary Fig. S10, with an exclusion of the assembles of *P*_*x*_ and *P*_*y*_ to avoid the PSP modes, a large-scale POPA is designed and fabricated (at least 5,826 nanopatches are excited according to the experimental results). The geometrical parameters are as follows: *W*_*s*_ = 100, *T*_*s*_ = 100, *H*_*s*_ = 700, *h* = 700, *T*_*p*_ = 100, *H*_*p*_ = 700, *R*_*p*_ = 180, *D*_*p*_ = 600, *P*_*x*_ = 1200 and *P*_*y*_ = 1100 in nm. The image of the fabricated sample under microscopy and the SEM image of the fabricated sample are shown in Fig. 6a and b, respectively. The details of the fabricated sample can be found in Section 6 of Supplementary Information.

The measurement setup is described in ref. 49. Based on the measured real-space image, the excitation area is over 56 λ in the *x-*axis and over 118 λ in the *y-*axis (Supplementary Fig. S14). It is thus impractical to obtain the solutions of such an electrically large structure directly with numerical simulations. As an alternative, a theoretical calculation based on the measured real-space image is developed to get the far-field results of the fabricated POPA (Supplementary Information Section 8).

Figure 6c shows the measured far-field emission pattern at 1540 nm with the theoretical one on its right side. The white lines in Fig. 6c indicate the far-field power distributions along two reference lines traversing the peak of the beam, as depicted in the insets of Fig. 6d and e. A reasonable agreement between the measured and theoretical beam shapes is observed. The measured and the theoretical power profiles along the two aforementioned reference lines are compared in Fig. 6d and e, respectively, where a beam scanning behavior is clearly observed as expected. Note that the actual phase distribution along the *y-*axis is very likely to deviate from an ideal quadratic phase distribution of a Gaussian beam. It is thus reasonable to believe that the deviation between the theoretical and measured profiles in Fig. 6e originates from the inaccurate *φ*_*n*_ (see the determination of *φ*_*n*_ in Supplementary Information Section 8).

The theoretical peak directivity of the fabricated POPA is plotted in the inset of Fig. 6e. It can be seen that a directivity of 38.7 dBi with a full width half maximum (FWHM) of 0.68° in the *x-*direction (Fig. 6d) is obtained near the telecommunication wavelength (1540 nm). With a wavelength-dependent projection angle and an ultra-small beam divergence angle, the proposed POPA has the potential to act as an integrated optical spectrometer^50^, a frequency-division multiplexer^51^ and is promising for the construction of wireless data nanolinks for plasmonic ICs^22^.

## Discussion

### Flat focusing POPA

In addition to beam-steerable POPAs, such a scheme can be extended to construct versatile POPAs with either large or medium scale. For demonstration, a flat focusing POPA which can act as a flat lens is designed. One of the unique features of this lens lies in that it focuses the in-plane propagating light at a focal point out of the plane of propagation. The potential applications of generating a focus include focal molography^52^, 3D interconnections in ICs^11^ and imaging.

**Figure 7:**
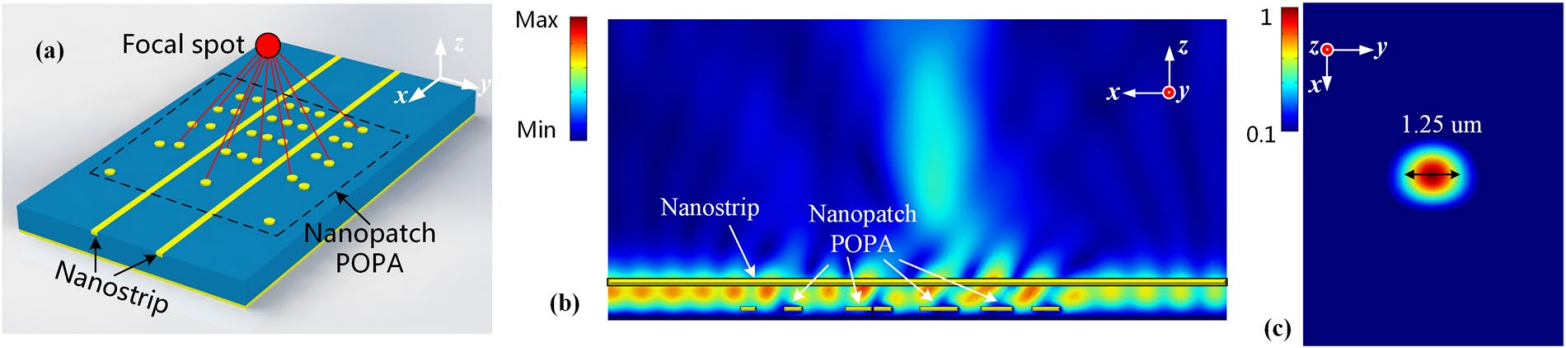
A flat focusing POPA. (**a)** Diagrammatic sketch showing the working mechanisms and the position of the focal spot. Two nanostrips are working together to harness those nanopatches. (**b)** E-field intensity distribution on the *xoz* plane with respect to which the array is symmetric. In this design, *H*_*p*_ = 100, *T*_*p*_ = 50, *R*_*p*_ = 140, *H*_*s*_ = 600, *T*_*s*_ = 100, *W*_*s*_ = 200 and *h* = 700, all in nm. (**c)** E-field intensity distribution on a cut-plane 3.6 μm above the polymer film, showing a diffraction-limited focal spot. The FWHM of the focal spot is 1.25 μm (wavelength:1550 nm).

To focus light at a certain spatial position, the position of each nanopatch in the POPA is designed to make the emissions from the nanopatches superpose in phase at a pre-defined location and is given by:

3 $${k}_{0}\sqrt{{(x-{x}_{0})}^{2}+{(y-{y}_{0})}^{2}+{(z-{z}_{0})}^{2}}+{k}_{g}(x-{x}_{0})=2n\pi $$

where (*x*_0_ *, y*_0_ *, z*_0_) and (*x*, *y*, *z*) stand for the coordinates of the pre-defined focal spot and that of the nanopatches, respectively and *k*_0_ is the free-space wavenumber. The diagram of the flat lens is depicted in Fig. 7a, where two nanostrips are aligned in parallel to handle a medium-scaled POPA. The near-field distributions in the *xoz* plane and at a cut-plane 3.6 μm above the polymer film are shown in Fig. 7b and c, respectively. As seen from Fig. 7c,a focal spot with a FWHM of 1.25 μm is formed by the coherent interference of the POPA.

## Conclusion and Outlook

In summary, a novel POPA has been reported and experimentally demonstrated in this paper. The beam of the proposed POPA is steerable and a high directivity can be achieved. The effects of the PSPs in the proposed POPA are investigated. The results indicate that the coupling between the fundamental Floquet mode and the PSPs has a significant impact on the radiation efficiency of the device and thus needs to be avoided in the design process. With the same topology, a medium-sized flat focusing POPA is also demonstrated which is capable of focusing in-plane propagating light into an out-of-plane spot.

The application prospects of the above described two POPAs are promising. For example, with the advance of the nano-fabrication technology, 3D interconnection is widely adopted for the design of silicon photonic chips^11^. For 3D interconnection, one will need to combine the in-plane propagation (propagating parallel to the surface of structure) with the radiation propagating out of plane^11^. The proposed flat focusing POPA which is capable of focusing the in-plane guided light at a focal point out of plane provides the potential for 3D interconnection in future plasmonic OICs. As another example, the superiority of the optical wireless nanolink constructed by nanoantennas in signal transmissions over highly lossy plasmonic waveguides has been demonstrated in ref. 22. The proposed beam-steerable POPA is a perfect candidate for the construction of such a datalink for plasmonic ICs. As the third example, the idea that improving laser efficiency with the aid of an antenna array has been successfully demonstrated in ref. 23. By replacing the SU-8 layer of the proposed beam-steerable POPA with gain assistant materials like cadmium sulphide (CdS), it is a potential candidate for the construction of high-efficiency plasmonic lasers. Moreover, according to the reciprocity principle, the proposed beam-steerable POPA also has the ability to precisely collect incoming light from a given direction, which is critical for some active devices like ultrafast photodetectors^53^ and integrated solar energy collectors^54^. Furthermore, the active-beam-scanning POPA can also be achieved by replacing the polymer film of the proposed beam-steerable POPA with refractive index controllable materials^55^.

## Methods

### Numerical simulations

The modal properties of the proposed nanostrip SPW are obtained with the eigenvalue solver from COMSOL Multiphysics, which is based on finite element method. The total calculation domain is 10λ in the *x*-axis and 6λ in the *y-*axis. The calculation domain is truncated by applying scattering boundary conditions in order to mimic a free space.

The demonstration of the capacitive coupling between the nanostrip and nanopatches in Fig. 2 and the flat focusing POPA in Fig. 7 are calculated with the frequency domain solver of COMSOL. In the simulation, a wave port is employed to excite the hybrid plasmonic mode of the nanostrip SPW.

For the design of the POPA with beam scanning ability in the far-field, the numerical results in Figs 4 and 5 are obtained by high frequency structure simulator (HFSS) which is based on finite element method.

The scattering response of the POPA under plane wave illumination in Fig. 5 was also obtained by HFSS. In the simulation, Floquet-bloch boundary is employed in a unit cell to make the simulated nanopatch a periodic one. Detailed simulation setups are depicted in Supplementary Fig. S11.

For all of the simulations, the adapative mesh refinement is enabled and the initial mesh sizes of the nanopatch and nanostrip are both 20 nm, and the initial mesh size of the silver film is 60 nm (wavelength: 1550 nm). The relative permittivity of silver is calculated using Drude model with *ε*_*inf*_ = 5, *ω*_*p*_ = 13.4 × 10^15^ rad/s and Γ = 1.12 × 10^14^ 1/s.

### Sample fabrication

In this work standard electron beam lithography (EBL) (Crestec CABL-9510C) and lift-off process are employed to fabricate the designed nanoantennas. The fabrication process is described in details as follows:

Firstly, a silver background layer (100 nm) is deposited onto the silicon wafer by using the thermal evaporator (Denton Vacuum DV-502A). Then a SU-8 layer (700 nm) is spin-coated onto the sliver layer and fully cured on a hot-plate at 160 °C. Then, a positive poly methyl methacrylate (PMMA) resist layer (500 nm) is spin-coated on the SU-8 layer and baked at 180 °C. The nano-patterns are fabricated on the PMMA film by EBL following by standard developing process. Prior to final silver deposition, a plasma treatment is performed on the SU-8 layer to improve the adhesion of silver to the SU-8 layer. A 100 nm silver film is then deposited on the sample by thermal evaporation. Finally, the nanostructure is achieved by a lift-off procedure.

### Experimental setup

The experimental setup is described in ref. 49. In the measurement, an infinite conjugate imaging system consisting of two lenses is employed to capture the real-space image of the fabricated sample. The real-space image provides a guideline for the alignment of the imaging system to the fabricated nanoantenna array. After the real-space image is recorded, an additional lens is inserted, and the imaging system turns into a far-field one.

## Electronic supplementary material

Supplementary Information

Video-S1

Video-S2
