# Supplementary material for: All-plasmonic Optical Phased Array Integrated on a Thin-film Platform
Source: Sci Rep. 2017 Aug 30;7:9959. doi: 10.1038/s41598-017-10398-8 (PMC5577131; doi:10.1038/s41598-017-10398-8)
Supplement: Supplementary file 1 — Supplementary Information [file 41598_2017_10398_MOESM1_ESM.pdf]

All-plasmonic Optical Phased Array Integrated on a Thin-film Platform

Yuan-Song Zeng[1], Shi-Wei Qu[1*], Bao-Jie Chen[2], and Chi Hou Chan[2]

[1]School of Electronic Engineering, University of Electronic Science and Technology of China (UESTC), Chengdu 611731, China

[2]State Key Laboratory of Millimeter Waves, Partner Laboratory in City University of Hong Kong, Kowloon, Hong Kong, China.

*e-mail: shiweiqu@uestc.edu.cn

S.1 Details about the nanostrip SPW under investigation 3
S.1.1 Evolution from a conventional nanostrip waveguide to the proposed SPW 3
S.1.2 Field distributions of the mode under investigation 5
S.1.3 Propagation distance of the proposed SPW 5
S.1.4 Details about the hybrid mode 6
S.1.5 Dependence of the propagation distance on nanostrip's width and position 9
S.1.6 Field distributions of the nanostrip array 10
S.1.7 Dependence of E-field intensity profiles upon the distance between nanostrips 11
S.2 Method of the calculation of the efficiency comparison in Fig. 5a 11
S.3 Calculated P_x and P_y when the interaction between PSPs and the fundamental Floquet mode occurs 12
S.4 Array response under plane wave illumination 12
S.5 Discussion on the red-shift of LSPs caused by LSP-PSP interaction 14
S.6 Details about the fabricated sample and the measurement 16
S.7 Real-space image of the experimentally characterized POPA 17
S.8 Theoretical method of obtaining the radiation of the experimentally characterized POPA based on the measured real-space image 18
(1) The single nanopatch radiation pattern 18
(2) $|A_m|$ 19
(3) φ_m 19
(4) $|A_n|$ 19
(5) φ_n 20
S.9 Measured and theoretical emission patterns of the fabricated POPA 20
S.10 Effects of the high order mode 21

S.1 Details about the nanostrip SPW under investigation

A mode formed by hybridizing the ss_b^0 mode of a nanostrip[1] and surface plasmon polaritons (SPPs) on a metal-dielectric interface is supported by this waveguide. The meaning of the nomenclature ss_b^0 for identifying the mode is as follows. The first two letters are used to identify the symmetry of the dominant transverse component (here, *y* component) with respect to the *z* and *y* axes, respectively, and '*s*' denotes a symmetric distribution. The subscript '*b*' means that this mode is a 'bound' mode while the superscript is used to track the extrema along the largest dimension (here, *y*-axis direction).

The features of this mode are summarized as follows.

S.1.1 Evolution from a conventional nanostrip waveguide to the proposed SPW

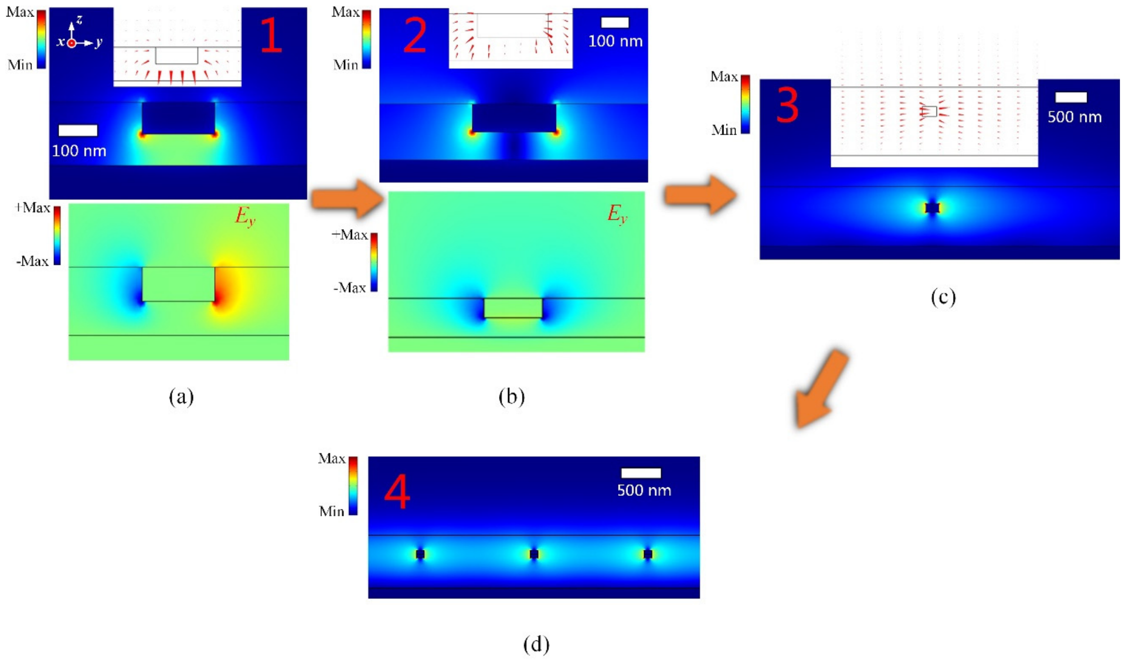

Fig. S1

Schematics showing the mode evolutions from the gap plasmon mode of a conventional nanostrip waveguide to the hybrid mode supported by the proposed waveguide. The total E-field intensity is plotted for all five waveguides. The insets in a-d show the E-field vector distributions and the lower panels in (**a**) and (**b**) show the distributions of E_y component. The detailed parameters of these waveguides are shown in Tab. S1. All the results are obtained at 1550 nm.

In Fig. S1, waveguide 1 is a conventional plasmonic nanostrip waveguide operating in the sa_b^0 mode of a nanostrip (E_z is the dominant transverse component). As seen from Fig. S1a, fields of a conventional nanostrip waveguide are tightly confined in the gap formed by the nanostrip and the silver film. This mode can be classified as a gap SPP mode. By increasing the width of the nanostrip, a new mode in which the SPPs at two metal corners decouple from each other emerges in waveguide 2. In addition, the symmetry of the E_y component experiences a flip. As seen from the lower panels of Fig. S1a-Fig. S1b, the E_y

component in waveguide 1 is asymmetric with respect to the *z* axis while it is symmetric in waveguide 2. The symmetry of the E_y component with respect to the *xoz* plane is vital for making a nanostrip array working coherently as a unity. This mode belongs to the corner SPP mode. Note that the corner SPPs in waveguide 2 exhibits a strong coupling with the plasmonic film. Waveguide 3 is acquired by decreasing the width of the nanostrip in waveguide 2 to 0.15 μm and lifting the nanostrip up to be 0.4 μm above the silver film to decouple from it. Decoupling the nanostrip from the silver film insures the single mode operation, which will be discussed later. The last figure marked as 'Waveguide 4' in Fig. S1 exhibits the results when multiple nanostrips are aligned in parallel.

Table S1. Detailed parameters of the nanostrip waveguides in Fig. S1.

waveguide number	*Hs*	*Ws*	*h*	*Ts*
1	100 nm	250 nm	200 nm	100 nm
2	100 nm	300 nm	200 nm	100 nm
3	400 nm	150 nm	700 nm	100 nm
4	400 nm	100 nm	700 nm	100 nm

S.1.2 Field distributions of the mode under investigation

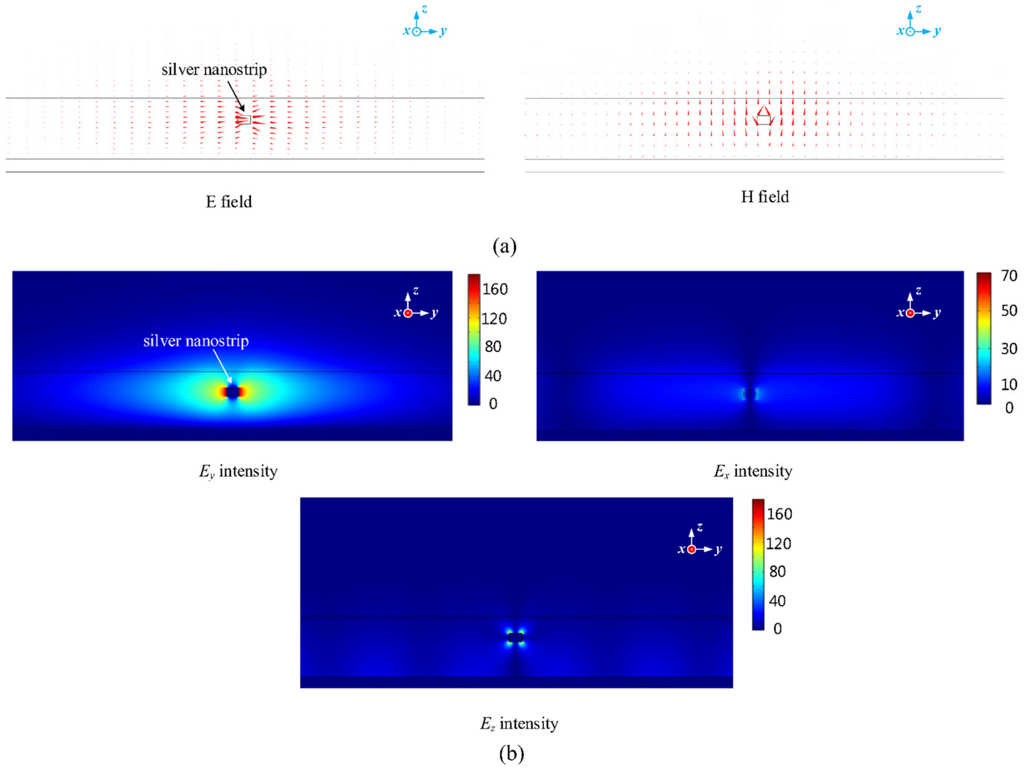

Fig. S2

Field distributions of the mode under investigation. (**a**) Vector plots of the distributions of electric fields and magnetic fields. (**b**) Intensity distributions of three E-field components. The parameters are as follows: H_s = 400, W_s = 200, T_s = 100 and h = 700 in nm. The results are obtained at a wavelength of 1550 nm.

S.1.3 Propagation distance of the proposed SPW

Table S2. Comparison between the proposed SPW and three conventional SPWs

Waveguide	Parameters	Propagation distance (μm)
Gap plasmon waveguide[2]	Width of the gap: 30 nm, thickness of the metal: 100 nm, immersed in silica	15
V-groove channel[3]	Opening angle: 20.4° , groove depth: 1 μm	45
Traditional nanostrip[4]	T_s = 50 nm, W_s = 200nm, h = H_s = 100 nm	13
Proposed SPW	T_s = 100 nm, W_s = 100 nm, h = 700 nm, H_s = 400 nm	264

*The results of the V-groove channel SPW are obtained at a wavelength of 1360 nm while the results for other three waveguides are obtained at 1550 nm

* All the waveguides are based on silver

* The calculation method of the modal size is from Ref. 5.

S.1.4 Details about the hybrid mode

Actually, two types of the hybridizations between the ss_b^0 mode and SPPs on the metal-dielectric interface are supported by the plasmonic waveguide under investigation. The field distributions of these two modes are shown in Fig. S3. As seen from Fig. S3, the real parts of their propagation constants are approximately equal. Compared to the right-sided one, the left-sided one exhibits much stronger coupling with the silver film via E_z component and its propagation distance is significantly shorter than the right-sided one (55 µm versus 97 µm). For convenience, in the following discussions, the left-sided one will be referred to as mode Type-I and the right-sided one as mode Type-II.

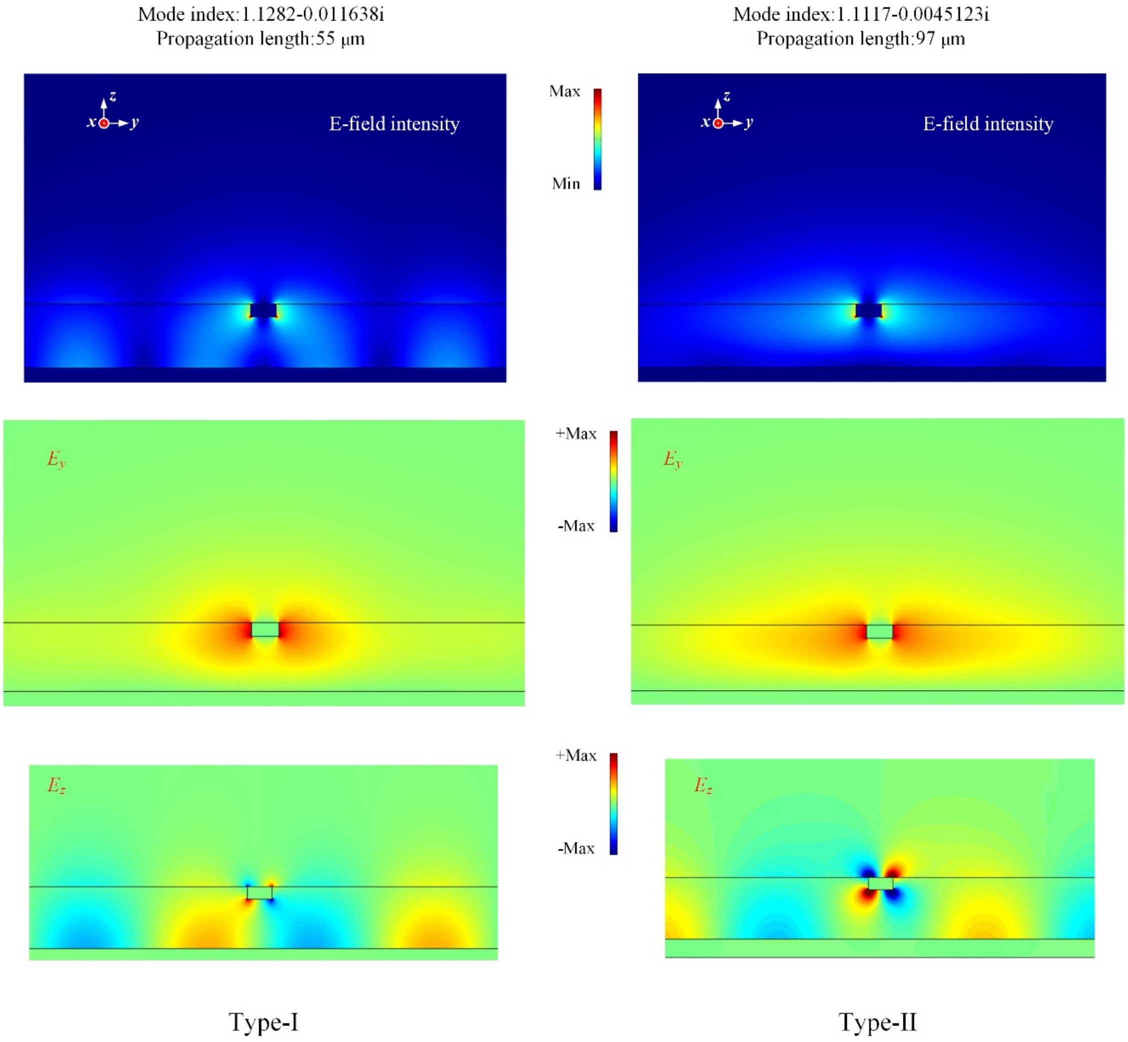

Fig. S3

Field distributions of two hybrid mode types. The parameters of the waveguide are as follows: $H_s = 400$, $W_s = 200$, $T_s = 100$ and $h = 500$ in nm and the results are obtained at a wavelength of 1550 nm.

For Type-II mode, although it is formed by hybridizing the ss_b^0 mode of a nanostrip[1] and surface plasmon polaritons (SPPs) on a metal-dielectric interface, the ss_b^0 mode and the SPPs are actually two uncoupled systems. The field distributions of the E_z component

for Type-I and Type-II modes are shown in the bottom of Fig. S3. From the phase states of the E_z fields confined on the tips of the nanostrip and in the vicinity of the silver film, it can be inferred that for Type-II mode, the displacement currents start from the positive charges on the tips of the nanostrip will not end in the negative charges on the silver film and vice versa. As there are no any other additional structures to sustain free charges, the displacement currents start from the positive charges on the tips of the nanostrip are supposed to end in the negative charges on the nanostrip again so that the current continuity equation can be satisfied. It means that for Type-II mode, the charge distributions on the tips of the nanostrip will follow the same principles determined by the ss_b^0 mode supported by an isolated nanostrip.

For further verification, E-field intensity distributions of the nanostrip waveguides with and without the silver film below the polymer layer are compared in Fig. S4. It can be seen that when the silver film is removed, the nanostrip itself indeed can sustain a mode with a similar modal field distribution. When the silver film is presented, the charges induced by the SPPs on the metal-dielectric interface distort the total field distributions and the hybridization with SPPs significantly reduces the propagation distance from 225 μm to 90 μm. The charge distributions of the Type-II mode in the *yoz* and the *xoy* plane are summarized in Fig. S5. Note that the E_y component of the SPPs also contributes to the total E_y fields of the hybrid mode and thus a slightly larger modal field expansion is observed for mode Type-II.

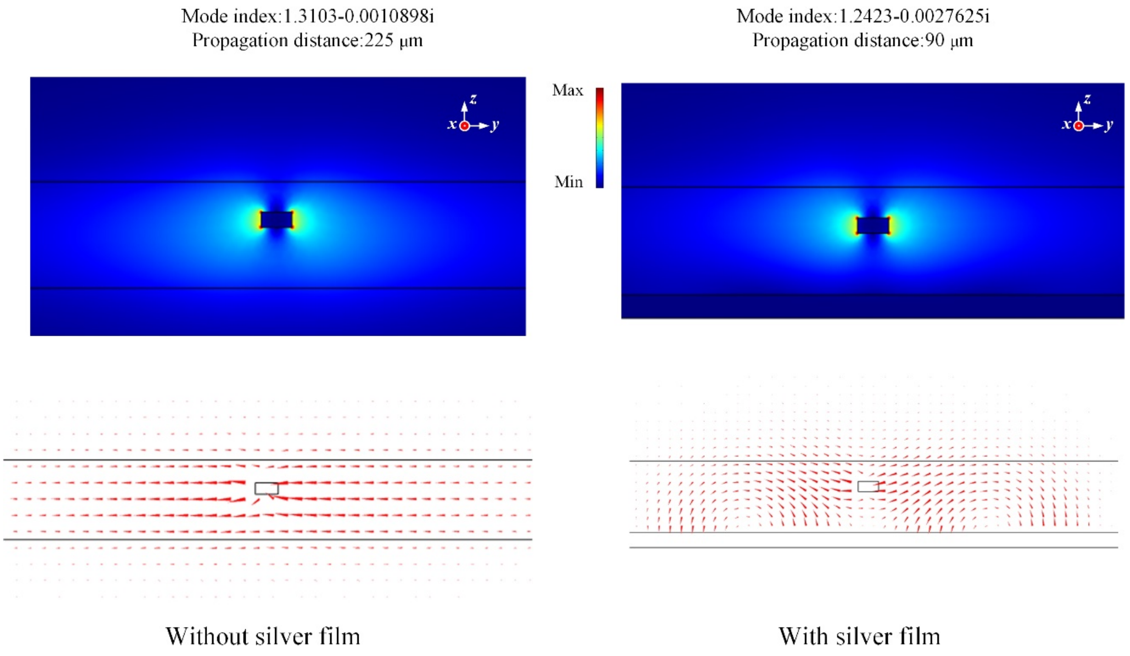

Fig. S4
Field distributions of the mode with or without the silver film beneath the polymer layer. The parameters are as follows: $H_s = 400$, $W_s = 200$, $T_s = 100$ and $h = 700$ in nm and the results are obtained at a wavelength of 1550 nm.

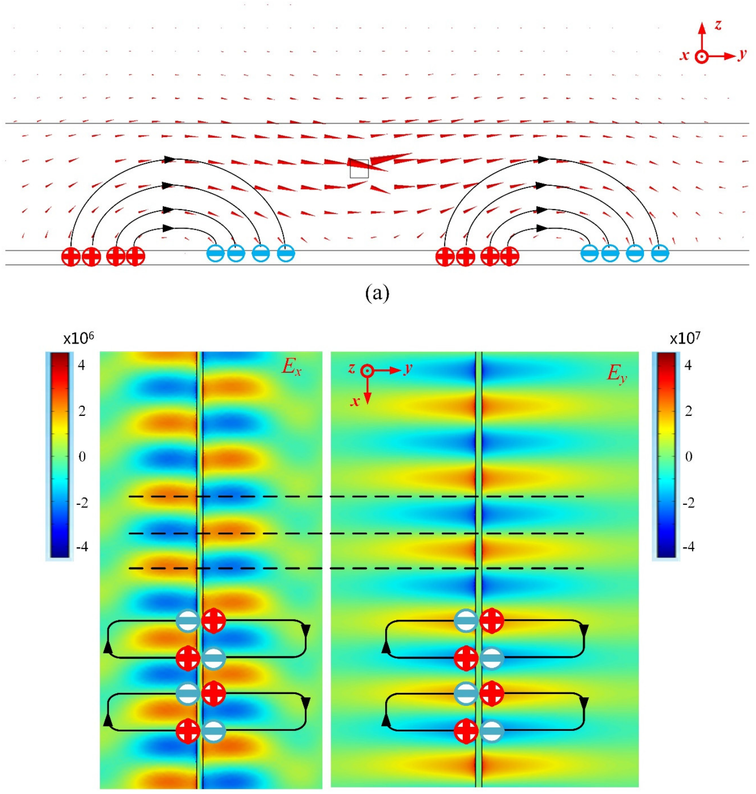

(a)

(b)

Fig. S5

Charge distributions of the Type-II mode at the *yoz* plane (**a**) and a cut-plane 400 nm above the silver film (**b**). The cones in (**a**) represent the E-field vectors. The E_x and E_y distributions at a cut-plane 400 nm above the silver film are depicted in (**b**). The dash line in (**b**) indicates a certain position at which the *x*-coordinate is the same in both figures. The parameters of the waveguide are: H_s = 400, W_s = 100, T_s = 100 and h = 700 in nm and the results are obtained at a wavelength of 1550 nm.

As has been mentioned above, the phase constants of modes Type-I and II are very close (1.1282 versus 1.1117) and they have very similar modal field distributions (their electric field intensities are both dominated by the *y* component). For practical applications, this is highly unfavorable. However, it is observed that when the height of the nanostrip is over 0.6 μm above the silver film, the Type-I mode no longer exists for a 0.3 μm wide nanostrip while the Type-II mode remains unaffected. In that case, the single mode operation can be assured. Moreover, when the nanostrip becomes wider, larger H_s is also required for single mode operation. This is not counter-intuitive as the extreme case where the width of the nanostrip goes to infinity corresponds to a standard MIM system. For a MIM system, two constituent metal blocks will decouple from each other if they are far away enough from each other. This decoupling is vital for practical applications and is referred to as ‘decouple from the silver film’ in the previously presented ‘Mode evolution’ section.

As the single mode operation can be provided by Type-II mode and the Type-II mode significantly outperforms the Type-I mode in terms of propagation distance, the Type-II mode is more likely to be the feeding-line mode of a POPA, which is the major goal of this

paper. Thus the second type of the hybrid mode will be of particular concern in this paper. The plotted field distributions in Fig. S2 are those of Type-II mode.

What is worth mentioning is that although a mode similar to Type-II mode can be sustained by the nanostrip alone, the silver film is still necessary because of the following reasons. Firstly, in practical fabrications, a substrate beneath the waveguide is required to support the whole structure. The existence of the silver film prevents the potential energy leakage into the substrate side. In addition, in plasmonic circuit designs, this metallic film can play the role of a ground plane in the circuits. Furthermore, the existence of the silver film improves the heat dissipation capacities of an integrated system.

S.1.5 Dependence of the propagation distance on nanostrip's width and position

Further investigations reveal that a cut-off height of the nanostrip for Type-II mode is found. Fig. S6 shows the dependence of the propagation distance on the height of the nanostrip above the silver film. Nanostrips with various widths are investigated in Fig. S6.

It can be seen that different cut-off heights are found for different nanostrip widths. Note that the ss_b^0 mode supported by an isolated nanostrip will not exhibit a cut-off without the silver film. It means that when the nanostrip and the silver film are too closely spaced, the influence of the charges on the nanostrip induced by SPPs on the silver film cannot be ignored so that the ss_b^0 mode cannot exist alone. These results further support the previous conclusion that the nanostrip and the silver film are uncoupled. It is also observed that when the nanostrip becomes wider, the cut-off height also increases. It is easy to be understood as the nanostrip becomes wider, the distance required for the existence of two uncoupled systems will increase as well.

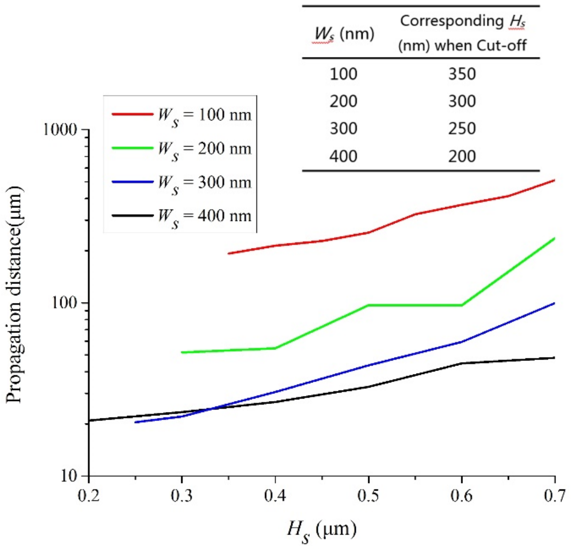

Fig. S6

Propagation distance versus the height of the nanostrip over the silver film. Various different widths of the nanostrip are considered. In this figure, T_s = 100 nm and $h = T_s + H_s$.

S.1.6 Field distributions of the nanostrip array

To diminish the significant lateral size mismatch with fiber, the nanostrips are aligned in parallel and the in-phase superposed mode is exploited. The E-field distributions of a single nanostrip and those of aligning two nanostrips in parallel are compared in Fig. S7. It can be seen that the field extension in lateral direction is equivalently magnified by the combination of two nanostrips, and no significant distortions are observed.

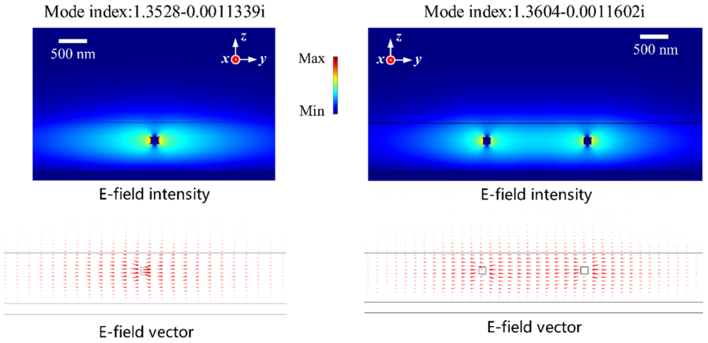

Fig. S7

Electric field distributions on a cross section of a nanostrip waveguide with W_s = 100, T_s = 100, H_s = 400 and h = 700 in nm. The left panel is for single nanostrip case while the right one shows the results when two nanostrips are aligned in parallel by a distance of 1.5 μm.

The results when 3, 5, 7 and 15 nanostrips are aligned together are shown in Fig. S8. With the increase of the amount of the nanostrips, the field intensities are getting more and more uniform.

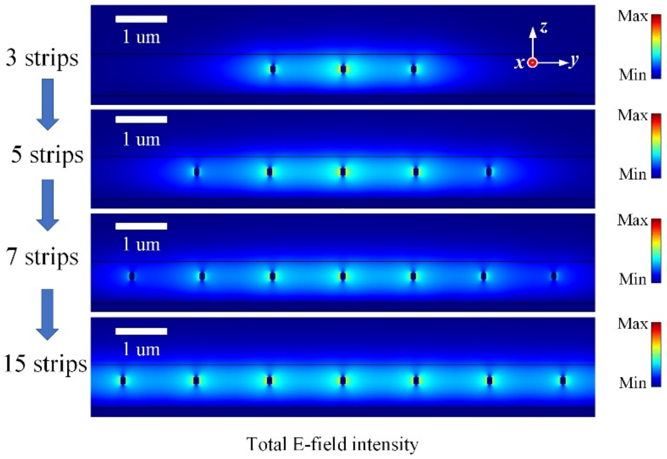

Fig. S8

Intensity distributions at a cross section of the nanostrip SPW array. The distance between the nanostrips is 1.5 μm while other parameters are the same as those in Fig. S7.

S.1.7 Dependence of E-field intensity profiles upon the distance between nanostrips

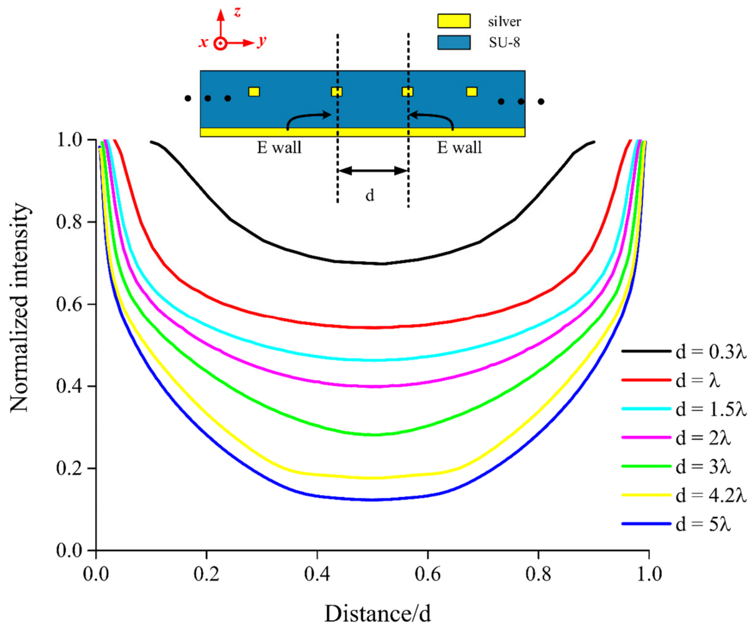

Fig. S9

Dependence of the E-field intensity profiles upon the distance between nanostrips. The intensity profiles are plotted along a reference line 450 nm above the silver film and in parallel with the *y* axis. In this figure, W_s = T_s =100, h = 700 and H_s = 400, all in nm.

S.2 Method of the calculation of the efficiency comparison in Fig. 5a

There are two parameters that give a measure of the ability of a phased array to concentrate electromagnetic power into a given direction. The first one is gain which is defined as:

$$G = 4\pi \frac{P(\theta,\varphi)}{P_{in}} \tag{1}$$

where P_{in} denotes the input power and $P(\theta,\varphi)$ is the radiated power at a specific direction. The second one is directivity which is given by:

$$D = 4\pi \frac{P(\theta,\varphi)}{P_r} \tag{2}$$

where P_r denotes the total radiated power. The ratio between them gives the radiation efficiency of a phased array which is given by:

$$\eta = \frac{P_r}{P_{in}} = G/D \tag{3}$$

In Fig. 5a, the radiation efficiency is calculated at the maximum radiation direction.

S.3 Calculated P_x and P_y when the interaction between PSPs and the fundamental Floquet mode occurs

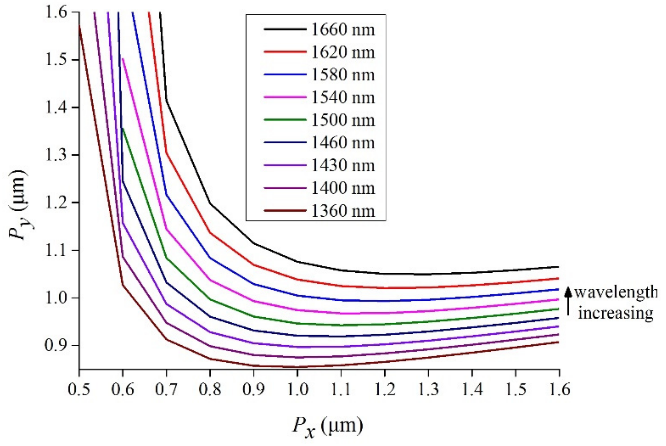

(a)

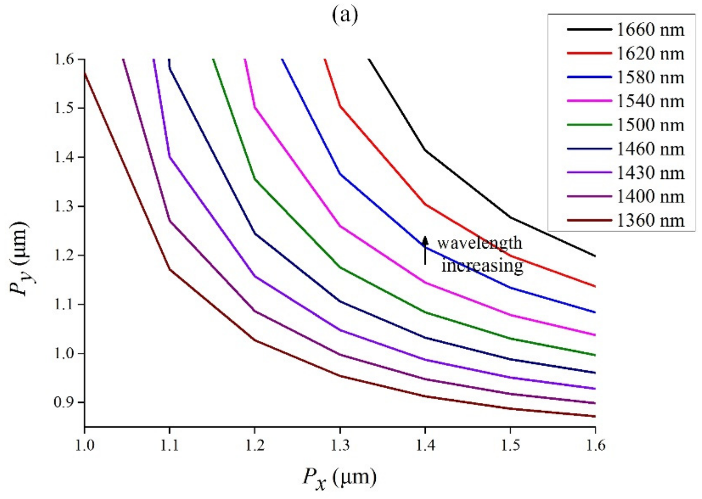

(b)

Fig. S10

Calculated P_x and P_y in the wavelength range between 1360nm and 1660nm when the coupling between the fundamental Floquet mode and PSP (0, ±1) mode (**a**) and PSP (-1, ±1) mode (**b**) happens. P_x and P_y are restricted in the range between 0.5 µm and 1.6 µm to avoid the grating lobes in the far-field.

S.4 Array response under plane wave illumination

In order to get a clear insight into the interaction between LSPs and PSPs in the POPA under investigation, the responses of the same nanopatch array under plane wave illumination are numerically studied. A schematic view of the simulation setup is shown in Fig. S*11*a. Floquet-bloch boundary conditions are applied in both the *x*-axis and *y*-axis directions and the incoming Floquet plane waves are imposed right above the array with normal incidence and *y*-polarized E-fields. Similar to what has been done in Ref. 6, one will need to fix the theoretical resonance wavelength of either PSPs or LSPs and alter the

remaining one. In this study, the case in which the geometric parameters of the nanopatch element are set to be fixed while the periodicity is altered with a step of 100nm, is investigated. The results are compared in Fig. S*11*b and Fig. S*11*c. In Fig. S*11*b, the theoretical resonant wavelengths of PSP (0,1) and (1,1) modes are plotted by continuous red and green lines, respectively. The field distributions of these modes are shown in Fig. S*11*c. The emergence of the PSPs can be readily recognized by the unique and strong E_z coupling with the silver film. These PSP modes are plotted with red and green box-dot in Fig. S*11*b. The simulated resonant wavelengths of the PSP modes agree quite well with the theoretical ones.

Besides, two trends can be evidently observed. Firstly, in Fig. S*11*b, the anti-crossing behavior between the LSPs and PSPs as previously reported[6] is not observed. Instead of exchanging their resonant positions in the frequency spectrum, the LSPs change their field distributions into another form after the crossing point (see Fig. S*11*b and Fig. S*11*c). The field distributions in "E-plane", i.e., the plane containing the dominant E-field component vector and the incident wave vector, before and after the crossing pointare compared in Fig. S*11*c along with the field distributions of the PSPs and an isolated nanopatch. Before the crossing point, the field distributions are close to those of an isolated nanopatch. After the crossing point, however, the mutual coupling between the adjacent nanopatches gets enhanced. These two modes are both considered as LSPs as their field distributions obviously distinguish them from a PSP mode. Another influence yielded by the enhanced mutual coupling is that after the crossing point, the resonant wavelength increases with the increase of the periodicity. However, the resonant wavelengths of the PSPs do not exhibit apparent deviations from the theoretical values, which is different from the anti-crossing type behavior where the resonant wavelengths of the PSPs and those of the LSPs are exchanged and the resonant wavelengths of the PSPs do not show a dependence upon the array spacing after the crossing point[6]. The special behavior here, is believed to result from the interaction of two resonant systems.

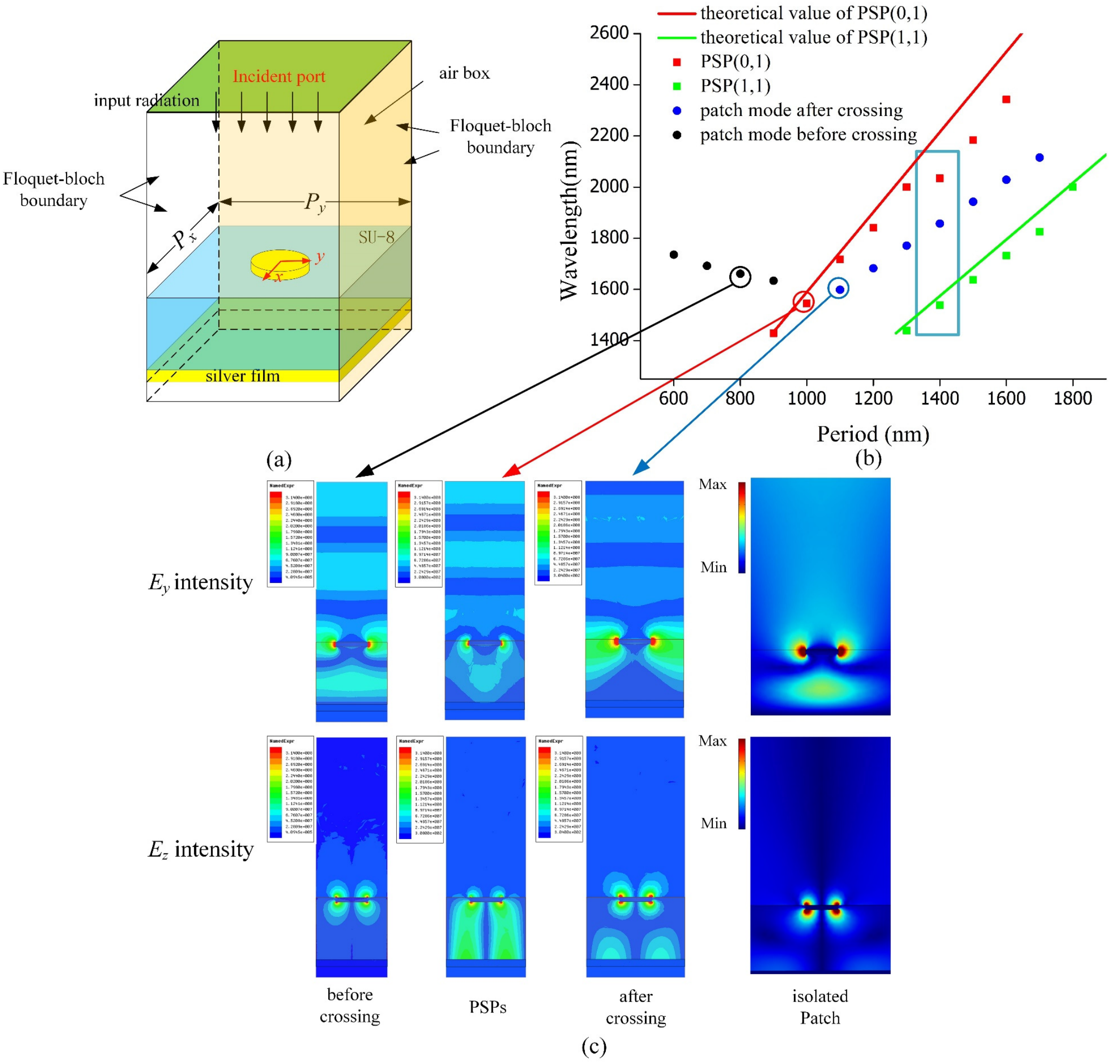

Fig. S11

Responses of investigated POPA under plane wave illumination. (**a**) Simulation setup. (**b**) The resonant wavelengths of the LSPs, PSP (0,1) and PSP (1,1) modes versus period. The red and green continuous lines show the theoretical predicted values of PSP (0,1) and (1,1) modes, respectively. The parameters are as follows: $T_p = 50$, $H_p = 650$, $R_p = 180$ and $h = 700$ in nm. (**c**) The field distributions at the resonant positions marked in (b) as well as those of an isolated nanopatch. From left to right: LSPs before crossing, PSPs (0,1) mode, LSPs after crossing, LSPs sustained by an isolated nanopatch. The upper panel shows the intensities of E_y and the lower panel for that of E_z. The first three figures share the same range of the colorbar. The cyan box marked out the case where the period is equal to 1400 nm and will be taken as a reference in the following discussions on the resonant frequency anomaly caused by the LSP-PSP interaction in the POPA excited by nanostrip SPWs.

S.5 Discussion on the red-shift of LSPs caused by LSP-PSP interaction

In the results under plane wave illumination, the LSPs sustained by the nanopatches exhibit a significant red shift after the crossing point due to their interaction with the PSPs. One may wonder whether the same situation stills holds for POPA fed by nanostrip SPWs.

Note that when the POPA is fed by nanostrip SPWs, the POPA is actually dealing with near-field in-plane light, instead of far-field out-of-plane light. For verification purpose, a POPA with P_x = 1.2, P_y = 1.38 and D_p = 0.69 in μm is investigated for numerical experiments. According to the results described in the main text, the resonant wavelengths of the PSP (0, ±1) and PSP (-1, ±1) modes are predicted to be 2070nm and 1500nm, respectively, and the resonant wavelength of the LSPs is approximately 1580nm. The above array configurations are chosen to well mimic the plane wave illumination case with P_x = P_y = 1400 nm (marked out by blue box in Fig. S11b). Under such an array configuration, for both the plane wave illumination case and the nanostrip excitation case, the LSPs are sandwiched by two PSP modes in the spectrum and the resonant wavelengths of the lower order PSPs and higher order PSPs are close.

The directivity, gain of the POPA fed by nanostrips as well as its radiation efficiency are shown in Fig. S12a and Fig. S12b. The emergence of the PSP (0, ±1) and PSP (-1, ±1) modes can be easily distinguished by both drops of radiation efficiency in Fig. S12b as well as the field distributions (not shown here for brevity). Note that in the case of plane wave illumination, the resonant wavelength of the LSPs goes down to approximately 1875 nm.

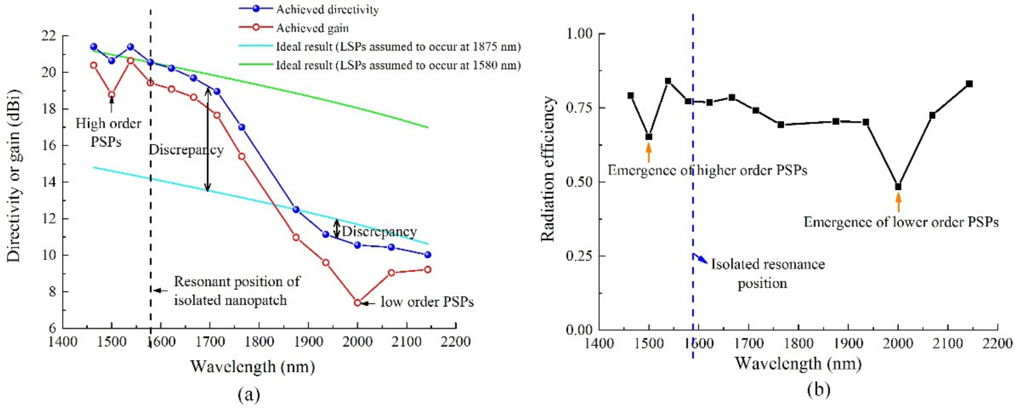

Fig. S12

(**a**) Achieved directivity, achieved gain curves and two ideal directivity results obtained by assuming that LSPs occur on either 1875 nm or 1580 nm in the POPA for numerical experiment. (**b**) Radiation efficiency of the POPA for numerical experiments.

Basically, the directivity of a phased array is the product of both the element radiation and array factor. In order to determine the contribution of each nanopatch element, one will need to distinguish them from that of an array factor. According to antenna theory[7], the directivity of a phased array is determined by:

$$D = \frac{4\pi S}{\lambda^2}\eta \tag{4}$$

In Eq. (4), S represents the geometrical area of the array aperture while η stands for the aperture efficiency. Specifically, for the near-field-excited POPA under investigation, the difference in η at different wavelengths is exclusively caused by the variation of the scan

angle. Hence, η is proportional to cos(θ) where θ represents for the scan angle with respect to the z axis. The scan angle θ is related to the array configuration as:

$$k_0 P_x \sin(\theta) = 2\pi(P_x - \lambda_g) / \lambda_g \qquad (5)$$

Assuming that the LSPs do experience a red-shift, and get pulled down to 1875 nm, the difference of the array factor gives the ideal result plotted as the cyan line in Fig. S12a, according to Eqs. (4) and (5). A clear discrepancy between the achieved directivity and ideal results is observed in Fig. S12a. Instead, with the assumption that the LSPs occur on 1580 nm, i.e., the resonant wavelength of the isolated nanopatch, a reasonable agreement can be found between the ideal directivity curve and the achieved one (green continuous line). This result indicates that in this design, the operating wavelength of the POPA is still determined by the dimensions of the nanopatch.

S.6 Details about the fabricated sample and the measurement

The sizes of each region on the fabricated sample are shown in Fig. S13. As the lithography cannot be performed on the edge of the sample, there is an un-patterned region there. This region is a grounded slab waveguide which plays the role of a transition between a fiber and the nanostrip SPW array.

Before the measurement of the fabricated sample, a transmission grating is used for the alignment of the imaging system. For the calibration of the measured far-field image of the fabricated sample, an additional grating emitter etched on a SU-8 slab waveguide is fabricated and the far-field image of the grating emitter is measured and adopted as a reference to characterize the far-field image of the fabricated sample.

All the presented far-field images of the fabricated sample in this work are measured without the help of a polarizer. Without saturating the detector when a y-polarized polarizer is applied, the cross-polarized light (x-polarized light) was not observed when a x-polarized polarizer is applied in the measurement. The above results indicate that when the camera is not saturated, the cross-polarized power is very small and out of the dynamic range of the camera. The polarization direction of the measured far-field results is consistent with the numerical simulation in which the radiation is dominated by the E_y component.

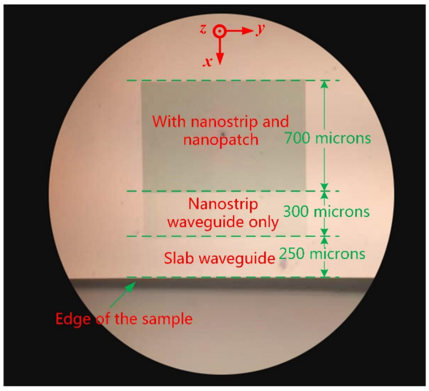

Fig. S13
Photograph of the fabricated sample under microscopy showing the size of each region.

S.7 Real-space image of the experimentally characterized POPA

As mentioned in the main text, a large area containing numerous nanostrips will be covered by a fiber. Determined by the Gaussian-beam nature of a fiber, the amplitude of the light guided by the nanostrip array is supposed to follow a tapering distribution along the y-axis direction. In other words, the amplitude gradually decreases from the center towards both sides. The real-space image of the fabricated sample supports the above inference.

The real-space image of the fabricated sample is shown in Fig. S*14*. As seen from the figure, the waveguide region is dark and a light-emitting region with one of its margins located on the boundary between the nanostrip-nanopatch region and the waveguide region appears after the waveguide region. A sharp boundary line between the waveguide region and the nanostrip-nanopatch region is observed. The depth of the light-emitting region gradually decreased from the middle towards both sides of the light-emitting region, fitting with a Gaussian distribution. In this figure, the excitation area covered by a fiber is roughly 182.96 μm in width and 86.85 μm in depth.

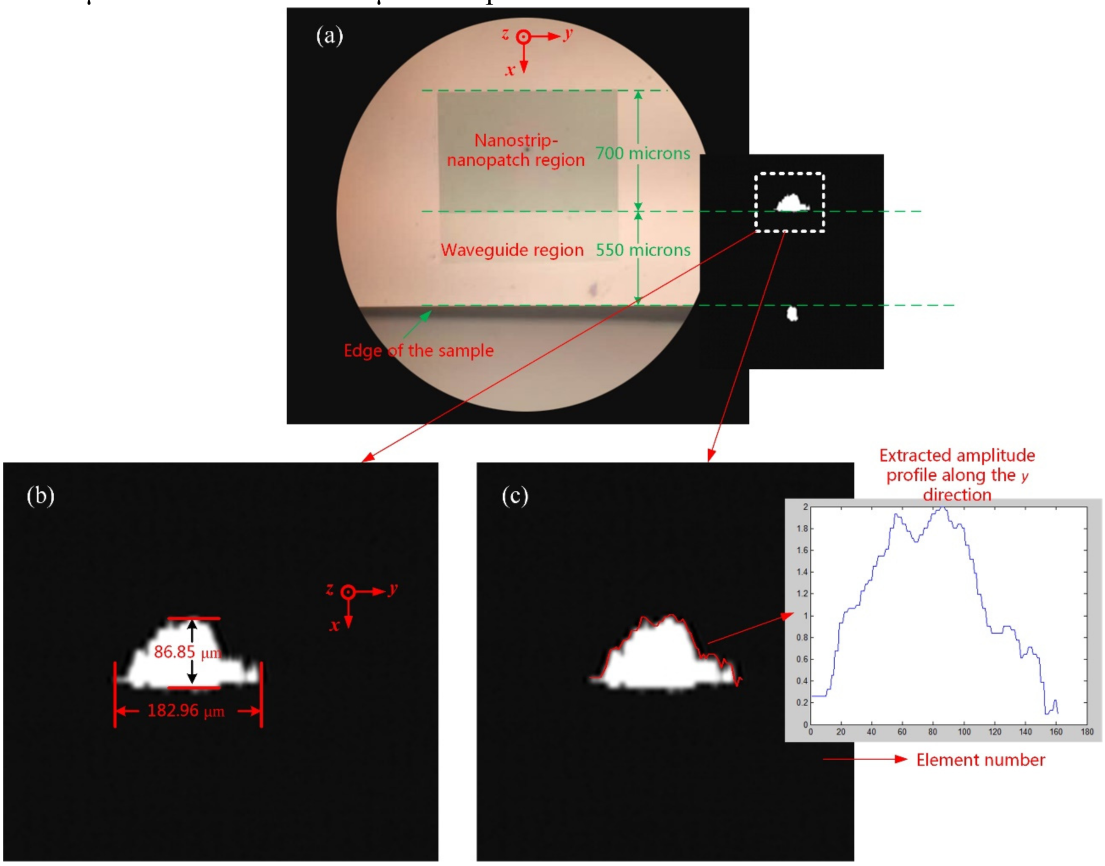

Fig. S14

(**a**) Real-space image of the fabricated POPA. (**b**) Sizes of the excitation area. (**c**) Extracted profile of the measured real-space image for the determination of $|A_n|$.

S.8 Theoretical method of obtaining the radiation of the experimentally characterized POPA based on the measured real-space image

The far-field radiation of the POPA can be expressed as:

$$\vec{E}_{\text{array}}(\theta,\varphi) = \vec{E}_{element}(\theta,\varphi) \times AF(\theta,\varphi) \tag{6}$$

where θ, φ represent the polar and azimuth angle, respectively. $\vec{E}_{element}(\theta,\varphi)$ is the far field pattern of a single nanopatch and $AF(\theta, \varphi)$ is the array factor. The array factor is given by:

$$AF = \sum_{m=1}^{M}\sum_{n=1}^{N} A_{mn} \exp[jk_0 \sin\theta(x_m \cos\varphi + y_n \sin\varphi)] \tag{7}$$

where (x_m, y_n) are the coordinates of the (m, n)-th nanopatch. A_{mn} is the complex excitation coefficient of the (m, n)-th nanopatch and contains both the amplitude and phase information:

$$A_{mn} = A_{mn} \exp(j\varphi_{mn}) \tag{8}$$

For the investigated POPA, apparently, A_{mn} satisfies:

$$\frac{A_{mn}}{A_{m0}} = \frac{A_{0n}}{A_{00}} \tag{9}$$

Therefore, AF can be written as [8]:

$$AF = \sum_{m=1}^{M} A_m \exp(jk_0 x_m \sin\theta\cos\varphi)\sum_{n=1}^{N} A_n \exp(jk_0 y_n \sin\theta\sin\varphi) \tag{10}$$

where $A_m = |A_m|\exp(j\varphi_m)$ and $A_n = |A_n|\exp(j\varphi_n)$ are the complex excitation coefficients along the x-axis and the y-axis direction, respectively. A_m, A_n and single nanopatch radiation are determined as follows:

(1) The single nanopatch radiation pattern

The radiation of a single nanopatch is obtained by numerical simulations. As depicted in Fig. S15a, in the simulations, an ideal current source was placed in proximity to the nanopatch to excite the fundamental mode of the nanopatch. The position of the ideal current source coincides with the peak of the E_z field of the nanopatch LSPs (Fig. 4a). The simulated far-field radiation pattern is plotted in Fig. S15.

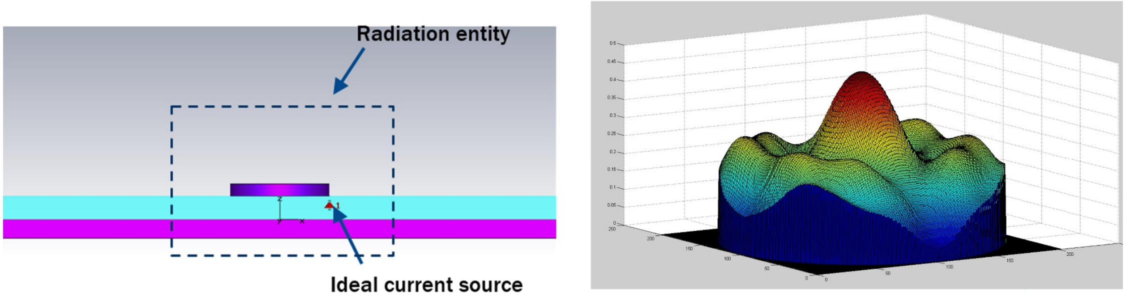

(a) (b)

Fig. S15

(**a**) Simulation setup of single nanopatch. (**b**) Obtained 3-dimentional emission pattern.

(2) $|A_m|$

The amplitude of the m-th nanopatch along the x-axis direction is given by:

$$|A_m| = \sqrt{c*(1-(|A_1|^2+|A_2|^2...+|A_{m-1}|^2)} \tag{11}$$

where c is the coupling coefficient between the nanopatch and the nanostrip. In Fig. S*16* the normalized amplitudes of the first 70 elements are plotted for a hypothetical c. An exponential decay can be clearly observed. In our calculations, the decay rate α is extracted from the full-wave simulations, i.e., from the E_y field intensity along the reference line 2 in Fig. 3c. Two different models with different amounts of nanopatches in the x-axis direction were simulated to verify this strategy. The first model with 24 nanopatches in the x-axis direction gives a decay rate of 0.303 dB/μm at 1540 nm while the second model with 36 nanopatches in the x-axis direction gives a close value of 0.289 dB/μm at the same wavelength. As the 36 nanopatches case corresponds to a lower terminal reflection and thus gives a better estimation of the practical decay rate, 0.289 db/μm is adopted as the decay rate in the theoretical calculations.

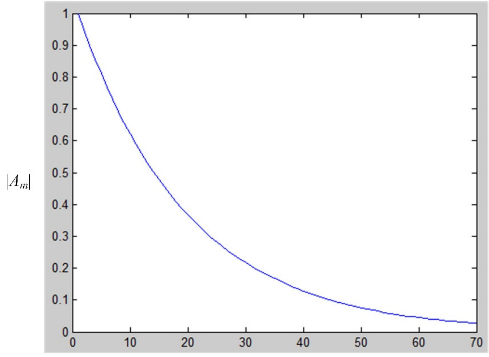

Fig. S16

Normalized $|A_m|$ versus element index, calculated according to Eq. (11).

Given a specific decay rate, 30 dB is taken as the criteria to determine the depth of the excitation, i.e., the amount of the nanopatches that are excited along the x-axis direction. Those with amplitudes 30 dB lower than the first nanopatch were not considered to contribute to the emissions. Taking the wavelength 1540 nm as an example, the depth of the excitation area is calculated to be 103.1 μm. The number of the nanopatches excited in the x-axis direction is therefore obtained by dividing 103.1 with 1.54, which is approximately 66.

(3) φ_m

The phase advance or phase lag along the x-axis direction is retrieved from the measured scan angle $\theta_{measured}$ and is given by:

$$\varphi_m = P_x \sin(\theta_{measured}) / \lambda \tag{12}$$

(4) $|A_n|$

$|A_n|$ is retrieved from the measured real-space image. As seen from Fig. S*14*, $|A_n|$ is extracted from the profile of the measured real-space image.

(5) φ_n

There is no suitable analytical model to describe the precise distribution of φ_n. However, considering the Gaussian-beam nature of a fiber, quadratic distribution can be used to provide an estimation of φ_n:

$$\varphi_n = a(y/\lambda)^2 \qquad \text{(in degrees)} \tag{13}$$

S.9 Measured and theoretical emission patterns of the fabricated POPA

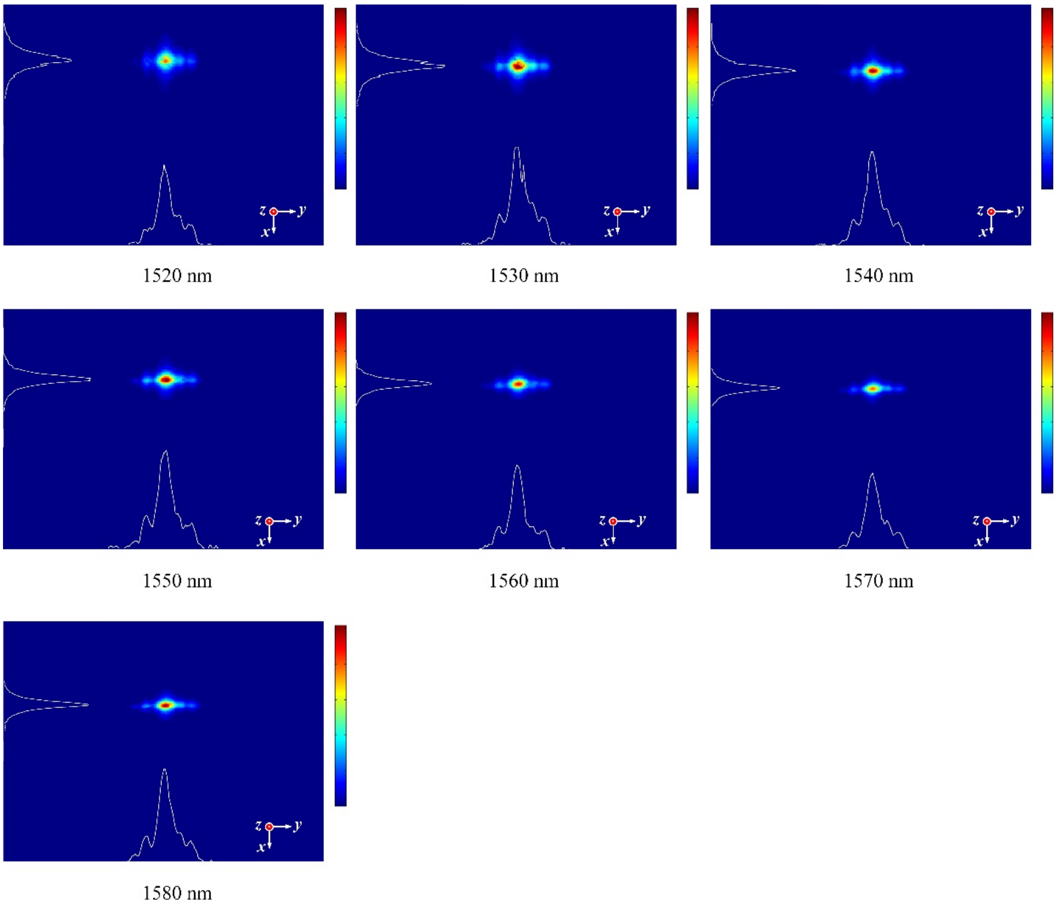

Fig. S17

Measured far-field power emission patterns of the fabricated POPA. The while lines indicate the power profiles along the two orthogonal lines traversing the peak of the beam.

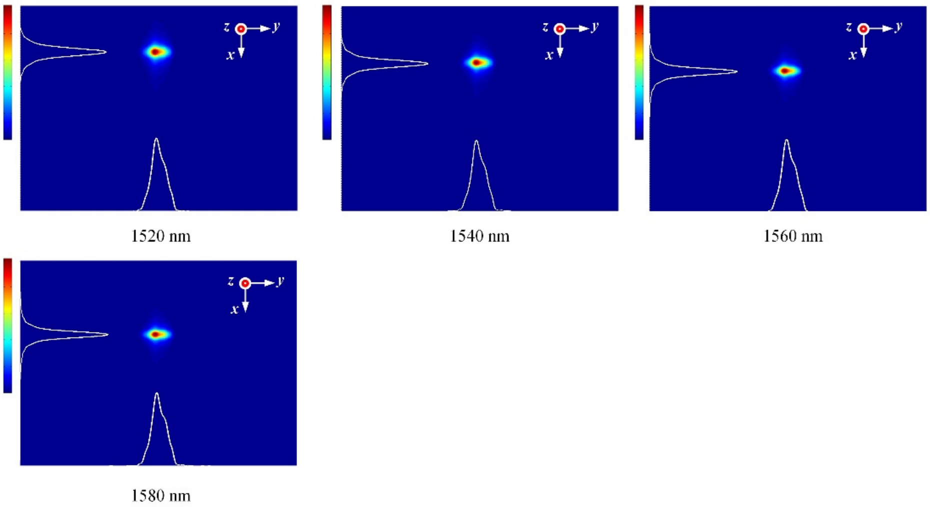

Fig. S18
Theoretically calculated emission patterns of the fabricated POPA with a = -0.125.

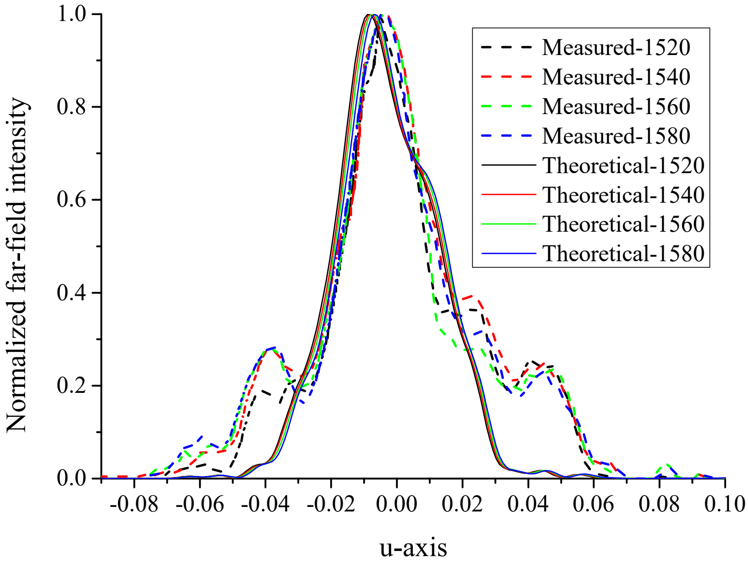

Fig. S19
Measured and theoretical power profiles along the reference line 2 in Fig. 6e with a = -0.125.

For both the theoretically calculated and the measured power profiles along the reference line 2, a weak dependence on the wavelength is observed.

S.10 Effects of the higher order mode

To investigate the effect of the higher-order mode on the antenna performance, nanopatch array operating in either the fundamental mode or the higher order mode is simulated. The models of the nanopatch array is shown in Fig. S20. The field distributions of the nanopatch arrays

operating in the fundamental mode and the first high order mode are depicted in the left and right panel of Fig. S21a, respectively. According to the analysis in Ref. 9, the state of the E_z component is selected to identify the order of the mode. It can be seen from Fig. R10a that when R_p = 200 nm, the nanopatches operate in its fundamental mode (o_{11}) and when R_p = 360 nm, the nanopatches operate in its first high order mode (e_{21}) at a wavelength of 1550nm[9].

The corresponding far-field radiation patterns of the nanopatch arrays operating in the fundamental o_{11} mode and the high order e_{21} mode are compared in Fig. S21b. It can be seen that a distortion of the radiation pattern is observed when the nanopatch array operates in high order mode. Moreover, the radiation efficiency drops from 79.18% of the fundamental mode to 59.13% of the high order mode. The results indicate that high order mode needs to be avoided in the design of the nanopatch array. In our simulations and experiments, an R_p of 180 nm is selected so that the nanopatches operate in the fundamental mode.

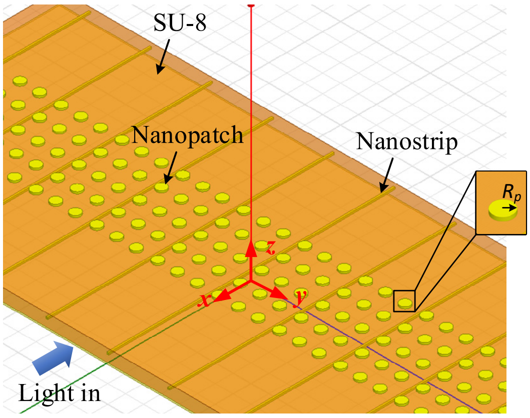

Figure S20. Geometric sketch of a nanopatch array for the investigation of the effects of the higher order mode. The simulation strategy described in the main text (Paragraph 2, Page 5) is used and a unit cell containing 5 x 3 nanopatches (5 in row, 3 in column). The geometrical parameters are as follows: H_s = 400, H_p = 700, h = 700, W_s = T_s = 100, D_p = 600, T_p = 100, P_x = 1200 and P_y = 1100 in nm.

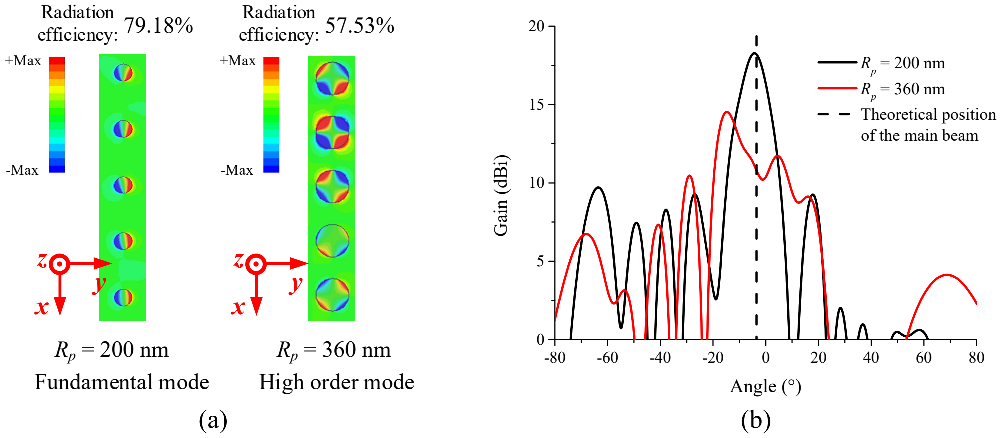

Figure S21. (a) Left panel: E_z component on the bottom surface of one column of the nanopatch array depicted in Fig. R9 when R_p = 200 nm. Right panel: E_z component on the bottom surface of one column of the nanopatch array depicted in Fig. S20 when R_p = 360 nm. (b) The radiation patterns on the *xoz* plane of the nanopatch array depicted in Fig. S20 with R_p = 200 nm and 360 nm. The results are obtained at the wavelength of 1550nm.

1. Berini, Pierre. "Plasmon-polariton waves guided by thin lossy metal films of finite width: Bound modes of asymmetric structures." *Physical Review B* 63.12 (2001): 125417.
2. Veronis, Georgios, and Shanhui Fan. "Guided subwavelength plasmonic mode supported by a slot in a thin metal film." *Optics letters* 30.24 (2005): 3359-3361.
3. Yan, Min, and Min Qiu. "Guided plasmon polariton at 2D metal corners." *JOSA B* 24.9 (2007): 2333-2342.
4. Hosseini, Amir, Hamid Nejati, and Yehia Massoud. "Design of a maximally flat optical low pass filter using plasmonic nanostrip waveguides." *Optics Express* 15.23 (2007): 15280-15286.
5. Oulton, Rupert F., et al. "A hybrid plasmonic waveguide for subwavelength confinement and long-range propagation." *Nature Photonics* 2.8 (2008): 496-500.
6. Chu, Yizhuo, and Kenneth B. Crozier. "Experimental study of the interaction between localized and propagating surface plasmons." *Optics letters* 34.3 (2009): 244-246.
7. Balanis, Constantine A., ed. *Modern antenna handbook*. John Wiley & Sons, 2011.
8. Mailloux, Robert J. *Phased array antenna handbook*. Vol. 2. Boston: Artech House, 2005.
9. *Minkowski, Fred, et al. "Resonant cavity modes of circular plasmonic patch nanoantennas." Applied Physics Letters 104.2 (2014): 021111.*
