# Supplementary figures and images for: All-plasmonic Optical Phased Array Integrated on a Thin-film Platform
Source: Sci Rep. 2017 Aug 30;7:9959. doi: 10.1038/s41598-017-10398-8 (PMC5577131; doi:10.1038/s41598-017-10398-8)

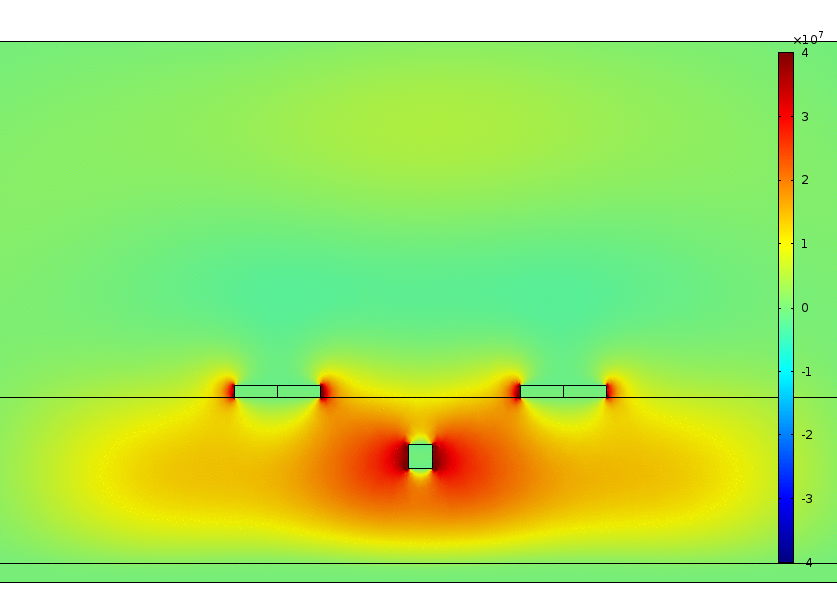

Supplement: Supplementary file 2 — Video-S1 [file 41598_2017_10398_MOESM2_ESM.gif]

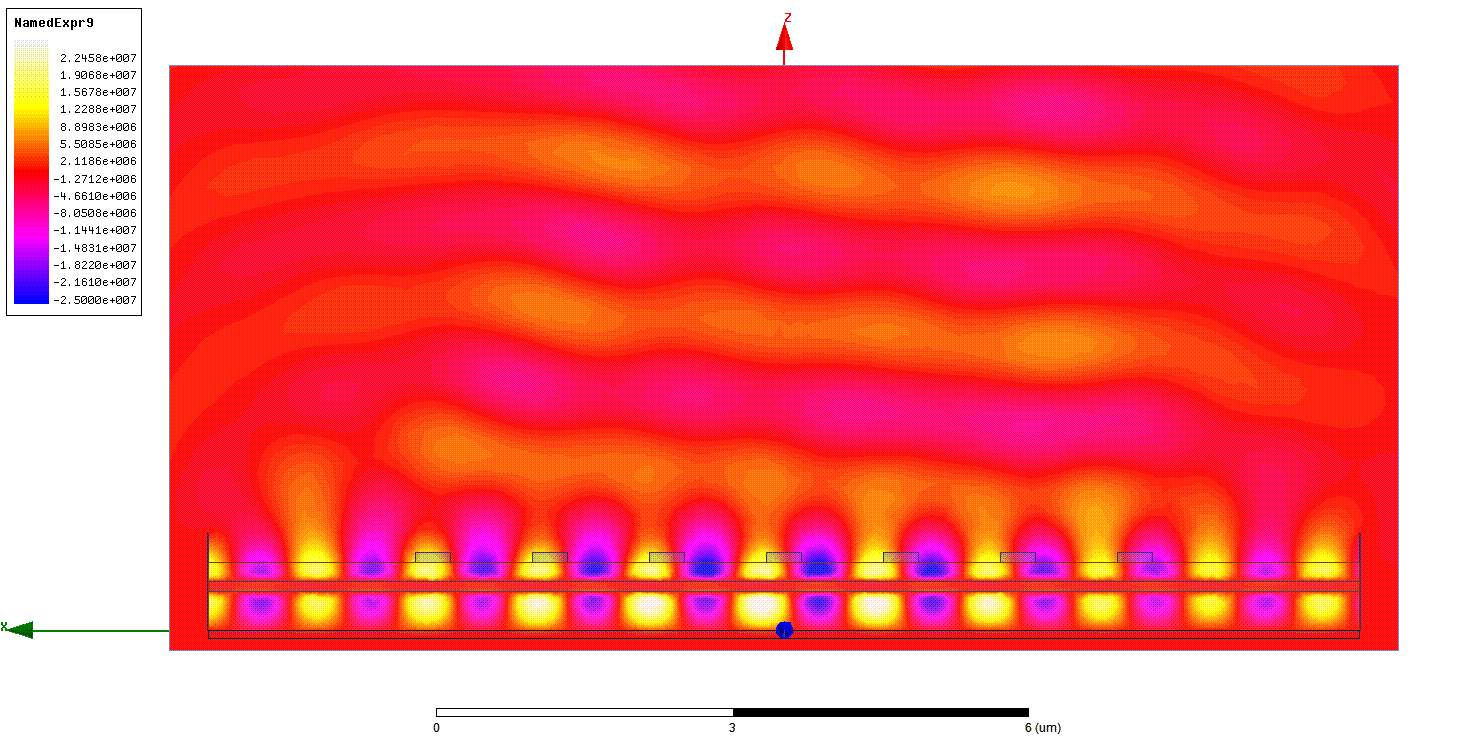

Supplement: Supplementary file 3 — Video-S2 [file 41598_2017_10398_MOESM3_ESM.gif]
